# Tailoring the Nutritional Composition of Italian Foods to the US Nutrition5k Dataset for Food Image Recognition: Challenges and a Comparative Analysis

**DOI:** 10.3390/nu16193339

**Published:** 2024-10-01

**Authors:** Rachele Bianco, Michela Marinoni, Sergio Coluccia, Giulia Carioni, Federica Fiori, Patrizia Gnagnarella, Valeria Edefonti, Maria Parpinel

**Affiliations:** 1 Department of Medicine—DMED, Università degli Studi di Udine, 33100 Udine, Italy; bianco.rachele@spes.uniud.it (R.B.); carioni.giulia@spes.uniud.it (G.C.); federica.fiori@uniud.it (F.F.); maria.parpinel@uniud.it (M.P.); 2 Branch of Medical Statistics, Biometry and Epidemiology “G. A. Maccacaro”, Department of Clinical Sciences and Community Health, Dipartimento di Eccellenza 2023–2027, Università degli Studi di Milano, 20133 Milan, Italy; michela.marinoni@unimi.it (M.M.); sergio.coluccia@unimi.it (S.C.); 3 Division of Epidemiology and Biostatistics, European Institute of Oncology, IRCCS, 20141 Milan, Italy; patrizia.gnagnarella@ieo.it; 4 Fondazione IRCCS Ca’ Granda Ospedale Maggiore Policlinico, 20122 Milan, Italy

**Keywords:** database harmonization, dish images, food composition database, food matching, manual data curation, missing imputation, nutritional composition of foods, nutrition, “Nutrition5k” dataset

## Abstract

Background: Training of machine learning algorithms on dish images collected in other countries requires possible sources of systematic discrepancies, including country-specific food composition databases (FCDBs), to be tackled. The US Nutrition5k project provides for ~5000 dish images and related dish- and ingredient-level information on mass, energy, and macronutrients from the US FCDB. The aim of this study is to (1) identify challenges/solutions in linking the nutritional composition of Italian foods with food images from Nutrition5k and (2) assess potential differences in nutrient content estimated across the Italian and US FCDBs and their determinants. Methods: After food matching, expert data curation, and handling of missing values, dish-level ingredients from Nutrition5k were integrated with the Italian-FCDB-specific nutritional composition (86 components); dish-specific nutrient content was calculated by summing the corresponding ingredient-specific nutritional values. Measures of agreement/difference were calculated between Italian- and US-FCDB-specific content of energy and macronutrients. Potential determinants of identified differences were investigated with multiple robust regression models. Results: Dishes showed a median mass of 145 g and included three ingredients in median. Energy, proteins, fats, and carbohydrates showed moderate-to-strong agreement between Italian- and US-FCDB-specific content; carbohydrates showed the worst performance, with the Italian FCDB providing smaller median values (median raw difference between the Italian and US FCDBs: −2.10 g). Regression models on dishes suggested a role for mass, number of ingredients, and presence of recreated recipes, alone or jointly with differential use of raw/cooked ingredients across the two FCDBs. Conclusions: In the era of machine learning approaches for food image recognition, manual data curation in the alignment of FCDBs is worth the effort.

## 1. Introduction

In the era of global nutrition research, the comparability of food composition databases (FCDBs) across culturally diverse countries is increasingly important [1,2,3,4,5]. In the past, several studies have similarly recognized that the harmonization of national/regional FCDBs allows individual and aggregate estimates of dietary intakes to be properly compared across multiple countries (e.g., [6,7,8]).

Information on dietary intake is still mostly collected through 24 h dietary recalls, food frequency questionnaires, and dietary records [9,10,11]. These traditional methods are affected by well-known issues (e.g., they are time-consuming, and data quality may be affected by consumers’ behavior) that can limit data collection [3,12]. Unprecedented opportunities to improve dietary assessment are offered by technological advancements that are reshaping the landscape of nutritional epidemiology [5]. For example, the integration of deep learning techniques (i.e., an artificial intelligence approach that teaches computers to process data in a way that is inspired by the human brain) in the domain of food image recognition is reported to be at the basis of innovative dietary assessment methods with higher accuracy and precision [13,14].

In the Nutrition5k project, a specific deep learning algorithm, deep convolutional neural networks (CNNs), was trained to predict the portion size and nutritional composition of food dishes obtained from US Google cafeterias [15]. The Nutrition5k project is also currently offering the first and largest open-access dataset for training deep learning algorithms on food images. As detailed information (RGB video streams, depth images, ingredient weights, and nutritional values from the US FCDB [16]) on almost 5000 real world food images is provided, Nutrition5k represents the natural framework to support the future development of a deep-learning-based dietary assessment tool able to: (1) recognize Italian foods and/or recipes and (2) estimate their nutrient content.

Since the accuracy and precision of such algorithms depend on the quality of metadata used for training, assessing potential differences in the nutritional composition of US and Italian foods is important. While investigating sources of differences in nutrient content across the two FCDBs, acknowledgement of natural variation in the amount of nutrients in foods over time and space [17,18,19] and country-specific variation in culinary traditions, portion sizes, and regional ingredients between the United States and Italy still support the need for a meticulous cross-country comparison of foods and nutrients. In addition, a standardized approach to handling missing values [17,20,21,22,23] is likely to reduce the risk of introducing additional bias in the analysis.

Within the framework of a widely used Italian FCDB, the Banca Dati di Composizione degli Alimenti (BDA) [21,24,25], the current study aims at:

(1) Elucidating challenges and solutions in linking the nutritional composition of Italian foods with food images from Nutrition5k;
(2) Assessing the presence of potential differences in nutrient content estimated across the Italian and US FCDBs and their determinants, within a comparative analysis.

This work holds promise not only for enhancing the accuracy of dietary assessments in Italy but also for paving the way toward a more globally applicable framework for leveraging deep learning in nutritional epidemiology.

## 2. Materials and Methods

The harmonization process was handled by a team of nutritionists with different expertise and seniority who shares the management of the Italian FCDB.

**Figure 1:**
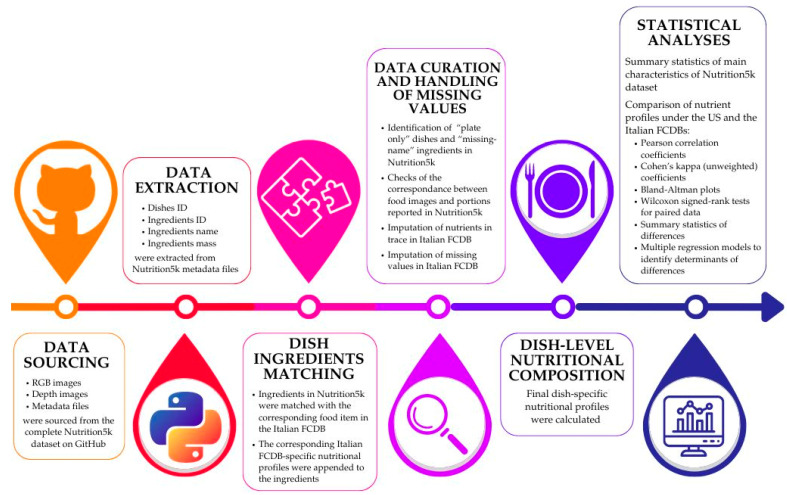
Comprehensive work plan related to research data acquisition and processing.

Figure 1 shows the workflow of the analysis, including data extraction, data curation, the matching between dish ingredients in Nutrition5k and food items in the Italian FCDB, the derivation of Italian-FCDB-specific nutritional compositions, and the statistical analyses needed to compare the nutrient content obtained for each dish under the Italian and US FCDBs. Additional details are provided in the following paragraphs.

### 2.1. Data Extraction and Preliminary Data Management

We downloaded metadata files of dishes (*dish_metadata_cafe1.csv*, *dish_metadata_cafe2.csv*) and ingredients (*ingredients_metadata.csv*) within the Nutrition5k project to extract nutritional information at both the dish- and ingredient-level. Within these files, each dish information is displayed in a single row and includes the following dish-level details: ID, total mass (corresponding to the mass in grams of the ration served in the cafeterias), number of ingredients, energy and macronutrient content (variables: *dish_id*, *total_mass*, *total_calories*, *total_fat*, *total_carb*, *total_protein*, and *num_ingrs*). Within each row, ingredient-level details are additionally displayed and include, for each ingredient used, the following: ID, name, mass (corresponding to mass of ingredients in the ration served), energy and macronutrient content (variables: *ingr_id*, *ingr_name*, *ingr_grams*, *ingr_calories*, *ingr_fat*, *ingr_carb*, and *ingr_protein*).

A single dish can be composed by one or more ingredients, and ingredients can be single foods or composite recipes. Nutritional values provided in Nutrition5k were automatically obtained by matching the corresponding ingredient with the US FCDB, i.e., the US Department of Agriculture food composition data [16].

From the metadata files, we extracted the following information: dish and ingredient IDs, ingredient name, and ingredient mass. We created separate rows for each ingredient within a dish by transforming the original dataset from the wide to the long format (Figure S1), to later link the Italian-FCDB-specific nutritional composition with the corresponding dish ingredient in Nutrition5k via food matching.

### 2.2. Exact and Indirect Matching of Ingredients with Their Nutritional Composition

We matched each ingredient from any Nutrition5k dish with the corresponding food item in the Italian FCDB and linked the corresponding Italian-FCDB-specific nutritional composition (expressed in terms of 86 components, including energy (2 components), water, alcohol, and macro- and micro-nutrients) to the dish- and ingredient-level information in Nutrition5k (expressed in terms of 477 components, including energy).

The linkage procedure requires that the definition of each component was checked across the Italian and US FCDBs [16,21]. A harmonization process was carried out for the following nutrients definitions in the Italian FCDB [21], which applied the corresponding US FCDB values [16]:

- *Available carbohydrates* = carbohydrate-by-difference—total dietary fiber;
- *Vitamin A components*:
  ○ β-carotene equivalents = 1 β-carotene + 0.5 α-carotene + 0.5 β-cryptoxanthin;
  ○ Retinol equivalent = retinol + 1/6 β-carotene equivalents;
- *Alpha-tocopherol equivalents* = α-tocopherol + 0.4 β-tocopherol + 0.1 γ-tocopherol + 0.01 δ-tocopherol + 0.3 α-tocotrienol + 0.05 β-tocotrienol + 0.01 γ-tocotrienol;
- *Short-chain saturated fatty acids*: butyric fatty acid (C4:0) + caproic fatty acid (C6:0) + caprylic fatty acid (C8:0) + capric fatty acid (C10:0).

Finally, since the variable “Other PolyUnsaturated Fatty Acids (PUFAs)” was present in the Italian FCDB but not in the US FCDB, we obtained the corresponding variable by summing the US-provided values for the single polyunsaturated fatty acids included in the US but not in the Italian FCDB.

The Italian FCDB contains nutritional values for each food item expressed per 100 g of the edible part; therefore, we expressed the Italian-FCDB-specific nutritional values on a mass basis for each ingredient in Nutrition5k. In addition, the nutrient content of dishes in Nutrition5k [15] was obtained via per-ingredient mass (i.e., incremental weight measured by a digital scale under the plate) and volume measurements (i.e., overhead depth data collected by an Intel RealSense D435 camera). Based on this information, we assumed that portions of ingredients and dishes provided in Nutrition5k referred to the cooked weight (e.g., pasta, meat, or cooked vegetables); therefore, when needed and available, for each ingredient, we referred to the cooked rather than the corresponding raw food item in the Italian FCDB to link nutritional values. When the cooked food item was not available in the Italian FCDB, we used the corresponding raw food item instead (e.g., *cooked salmon* in the US FCDB matched with (raw) *salmon* in the Italian FCDB).

We identified five matching criteria [26] between the Nutrition5k-specific ingredients and Italian-FCDB-specific food items, which are described in detail hereafter.

#### 2.2.1. Exact Matching between Ingredients in Nutrition5k and Food Items in the Italian FCDB

The matching was exact when: (1) the same name was available for the ingredient in Nutrition5k and the food item in the Italian FCDB (e.g., *eggplant–melanzane*, *salmon–salmone*, or *butter–burro*); (2) names were formally different, but it was possible to precisely identify the ingredient as the food available in the Italian FCDB (e.g., *celery root–celeriac*, *green beans–French beans*, or *cookies–biscuits*).

When an exact match was not possible, separate indirect matching strategies were adopted, as described in the following paragraphs.

#### 2.2.2. Indirect Matching: Similarity between Ingredients in Nutrition5k and Food Items in the Italian FCDB

When the Italian FCDB showed a food item with a similar definition, appearance, description, or nutritional composition, we considered it as a substitute of the Nutrition5k ingredient. When more than one food item in the Italian FCDB was suitable, we selected the one that was closest to that in Nutrition5k in terms of calories, macronutrients, and water content.

#### 2.2.3. Indirect Matching: Dish Ingredients Present in Nutrition5k Were Missing Food Items in the Italian FCDB

When a food ingredient from Nutrition5k was missing in the Italian FCDB and no suitable Italian alternatives were available, food items from the US FCDB were considered in the harmonization process. To identify the most appropriate food item for matching, we checked the nutritional composition of the closest US FCDB food items.

#### 2.2.4. Indirect Matching: Dish Ingredients Present in Nutrition5k Were Too Generic for Matching: Mean Values of the Corresponding Nutrients

When an ingredient in Nutrition5k was too generic (e.g., *berries*), we accounted for potential variability in food images (e.g., *cheese* could refer to cheddar cheese in a dish and to mozzarella cheese in another one) by identifying a group of potentially matching foods in the Italian FCDB. We then calculated the mean value of each nutrient associated with each Italian-FCDB-specific food item.

#### 2.2.5. Indirect Matching: Single Dish Ingredients in Nutrition5k Were Composite Recipes

When ingredients in the Nutrition5k dataset were composite recipes not available in the Italian FCDB, we created new recipes and adopted them as dish ingredients in Nutrition5k. Recipes were formulated by first using the Italian food atlas (i.e., “Atlante Fotografico Alimentare—uno strumento per le indagini nutrizionali”) [27] and, in its absence, the most followed cooking websites in Italy [28,29]. When the ingredient in Nutrition5k was a generic recipe (e.g., *tuna salad* or *pasta salad*) or the recipe was very unusual in Italy (e.g., *coleslaw* or *succotash*), we used dish images to better mimic the original recipes in Nutrition5k.

### 2.3. Manual Data Curation

#### 2.3.1. “Plate Only” and “Missing-Name Ingredients”

When a “plate only” dish was identified in Nutrition5k, the corresponding dish was removed from the dataset after assessing that the plate was empty. When an ingredient was indicated with its number but the corresponding name was missing in the dish-level files (*dish_metadata_cafe1.csv*, *dish_metadata_cafe2.csv*) (“missing-name ingredients” from now onwards), we used either the additional ingredients file (*ingredients_metadata.csv*) or the dish images to identify it.

#### 2.3.2. Ingredients Portion: Checks

We checked the summary statistics (minimum, 1st quartile, median, 3rd quartile, mean, standard deviation, and maximum values) of mass across all ingredients in Nutrition5k. When the maximum values were extremely high, we used dish images to identify the most likely portion and modified the mass accordingly.

#### 2.3.3. Nutrients in Trace

The Italian FCDB flags nutrients in trace amounts (i.e., present in such small quantities that cannot be measured adequately) with a specific code. The criterion to define amounts of nutrients as traces is <0.6, <0.06, or <0.006 depending on the analytical method used [17]. Therefore, we imputed trace values with 0.5, 0.05, or 0.005 according to the methodology proposed by Greenfield and Southgate [17]. For example, we indicated a retinol trace content <0.6 μg as 0.5 μg, a vitamin D trace content <0.06 μg as 0.05 μg, a tocopherol trace content <0.006 mg as 0.005 mg, and so on for each nutrient-specific criterion.

### 2.4. Missing Values

Several food items in the Italian or US FCDBs showed missing values for some nutritional components. To increase the comparability between the two FCDBs, we handled missing values by using a standardized approach [17,24], as described below:

- *Imputation by similar food items*: missing values were replaced with other values based on a similar food item (e.g., values for blueberries used for raspberries), or another form of the same food (e.g., values for “boiled” used for “steamed”);
- *Imputation by calculation*: missing values were imputed by calculation from incomplete or partial analyses of a food (e.g., carbohydrates or fats by difference, or chloride calculated from the value for sodium);
- *Imputation by assumption*: when the source or origin of the values may be referred to as “assumed” or “presumed” zero (e.g., vitamin B12 in vegetables), missing values were replaced with zero;
- *Imputation by recipe calculation*: missing values were substituted with values derived from recipes, calculated from the nutrient content of the ingredients and corrected for preparation factors (i.e., yield and retention factors);
- *Imputation by borrowed values*: when original sources were adequate, missing values were replaced with values taken from other tables and databases, including FCDBs from the USA, UK, Denmark, France, and New Zealand [16,22,23,30,31]. In some cases, the borrowed values were adapted to the different macronutrient content (e.g., calculation based on a reference profile for individual fatty acids, and/or individual soluble carbohydrates, and/or individual amino acids).

### 2.5. Dish-Level Nutritional Composition

After dishes and ingredients (including mass) were checked and missing values were imputed, final dish-specific nutritional information including mass, energy, macro- and micro-nutrient content (86 components plus mass) was calculated by summing the corresponding nutrient content for each dish.

### 2.6. Statistical Analysis

We calculated the summary statistics (minimum, 1st quartile, median, 3rd quartile, mean, standard deviation, and maximum values) for the distributions of the number of ingredients, mass, energy (here expressed in kcal), and the remaining 85 components across all dishes.

We also compared energy and macro-nutrients (i.e., proteins, fats, and carbohydrates) derived from the Italian FCDB and those indicated in the metadata files of Nutrition5k. The comparison was restricted to energy and macronutrients, because this is the only information provided in Nutrition5k; the comparison accounted for the correction of mass values described in Methods, Section 2.3.2, to avoid systematically inflating the difference with a material error already fixed. As the corresponding nutrient content was derived from the US FCDB, we herein refer to this comparison as Italian FCDB–US FCDB. The comparison was carried out by targeting the following measures of agreement or differences between the two FCDBs:

1. Scatterplot of each nutrient’s distributions under the two FCDBs, Pearson correlation coefficients, and hypothesis testing on the correlation coefficients;
2. Percentages of agreement on the classification of dishes into quintiles for each nutrient, and Cohen’s kappa (unweighted) coefficient for each nutrient to take into account the possibility of agreement occurring by chance; the cut-offs for quintiles were separately calculated on Nutrition5k and the Italian FCDB; interpretation of Cohen’s kappa results followed stricter criteria used in a recent publication [32]: 0.01–0.39 as none to slight, 0.40–0.59 as weak, 0.60–0.79 as moderate, and 0.80–1.00 as strong to very strong agreement;
3. Bland–Altman plot for each nutrient and corresponding 95% limits of agreement;
4. (Raw, absolute) differences between nutrients calculated with the Italian and US FCDBs (i.e., nutrients in the Italian FCDB–nutrients in the US FCDB), summary statistics of the difference distributions (minimum, 1st quartile, median, 3rd quartile, mean, standard deviation, and maximum values), kernel density estimation plots of the difference distributions, Kolmogorov–Smirnov normality test on the difference distributions (as the huge number of available dishes prevented us from using the Shapiro–Wilk method), and Wilcoxon signed-rank test for paired data for each nutrient (as the normality assumption was not satisfied for any of the investigated nutrients);
5. Differences in absolute values between nutrients calculated with the Italian and US FCDBs, to identify the nutrient-specific top 25 dishes showing the most extreme differences, regardless of the sign.

To further investigate potential determinants of disagreement between the two FCDBs, we fitted a series of ordinary least-squares multiple regression models including the difference between energy/macronutrient content as the dependent variable and the following independent variables as main effects: (1) dish mass (continuous); (2) number of ingredients per dish (discrete); (3) presence of recreated recipes in each dish (categorical, with “no” (reference category), “yes” categories); and (4) differential use of raw and cooked ingredients in each dish (categorical, with “no difference” (reference category), “one ingredient”, and “more than one ingredient” categories). The interaction term between the two categorical variables (differential use of raw and cooked ingredients and presence of recreated recipes in each dish) was added based on the nutritionists’ knowledge. Violations of ordinary least-squares model assumptions suggested using the robust MM estimator [33] in the final analyses.

All statistical tests were two-sided, with a statistical significance level of α = 0.05. Python (version 3.10) [34] was used for data structure management and curation, and the R statistical software (version 4.3.2) for the statistical analysis [35], with the corresponding packages [36,37,38,39] for robust linear models, robust ANOVA tests, and Wald tests for multiple coefficients (robust F-test) [40]. Food matching was performed using Microsoft Excel©, (version 2016).

## 3. Results

### 3.1. Data Curation of Dishes and Dish Ingredients in Nutrition5k

A total of 5006 dishes (4768 from the *café 1* and 238 from the *café 2* file) and 249 different ingredients among 28,455 total ingredients (i.e., total number of ingredients used over available dishes, with the same ingredient counted as many times as used) were available from the Nutrition5k datasets *café 1* and *café 2*.

We removed “plate only” (one item, appearing twice) from the total dishes, and this left us with 5004 dishes and 248 different ingredients. After matching the missing-name ingredients (1 item with no name appearing 110 times) with the additional ingredients in *ingredients_metadata.csv*, we regained 6 different ingredients appearing 79 times. We also manually investigated images of missing-name ingredients to regain two additional ingredients originally named as “deprecated” in *ingredients_metadata.csv*. These ingredients, watermelon and apples with peel (31 times recovered), were identified and manually assigned to former missing-name ingredients. This allowed us to impute all of the eight missing-name ingredients. In the end, 255 different types of ingredients were identified over a total of 28,453 total ingredients.

### 3.2. Exact and Indirect Matching between Ingredients in Nutrition5k and Food Items in the Italian FCDB

The Italian food composition data were matched to each of the 255 different ingredients available in Nutrition5k via exact or indirect food matching (details in the Section 2, Section 2.2.1, Section 2.2.2, Section 2.2.3, Section 2.2.4 and Section 2.2.5). Of them, 177 (~69%, corresponding to a total of 23,834 ingredients, ~84%) matched the corresponding Italian FCDB food items exactly, regardless of the official name used across the two FCDBs.

**Figure 2:**
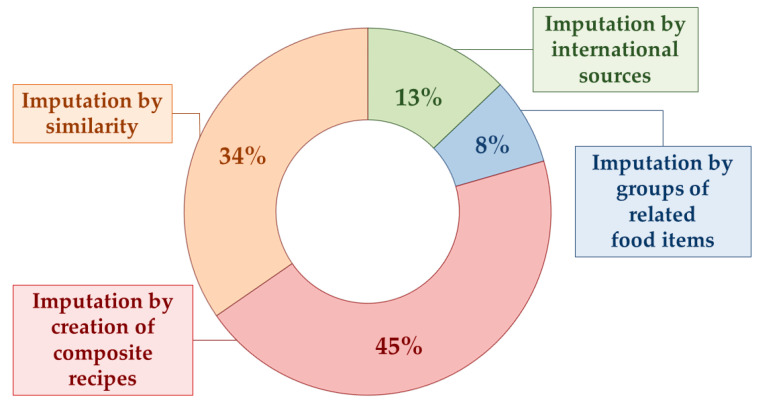
Indirect matching: imputation strategies and related frequencies.

Indirect matching involved 78 different ingredients (4619 total ingredients), as shown in Figure 2 and discussed in the following paragraphs. Ingredients from each category are shown in detail in Figures S2–S5.

#### 3.2.1. Indirect Matching: Similarity between Ingredients in Nutrition5k and Food Items in the Italian FCDB

Twenty-seven dish ingredients in Nutrition5k were formally unavailable in the Italian FCDB but could be replaced with their most similar food items (27/78~34% of total indirect matching procedures) (Table S1). The ingredient “*pepperoni*” is a user-friendly example: since it is not available in the Italian FCDB, we examined all the food items with a similar aspect and composition, and we chose a salami made of both beef and pork meat as the most similar ingredient.

#### 3.2.2. Indirect Matching: Dish Ingredients Present in Nutrition5k Were Missing Food Items in the Italian FCDB

Ten ingredients in Nutrition5k did not have any appropriate substitute among the Italian FCDB food items; they were retrieved in the US FCDB (10/78~13% of total indirect matching procedures) (Table S2). For instance, ketchup and barbecue sauce do not exist in the Italian FCDB; in the absence of suitable alternatives, we used the related items “*Catsup*” and “*Sauce*, *barbecue*” from the US FCDB.

#### 3.2.3. Indirect Matching: Dish Ingredients Present in Nutrition5k Were Too Generic for Matching: Mean Values of the Corresponding Nutrients

The nutritional values of six dish ingredients with generic names in Nutrition5k (6/78~8% of the total indirect matching procedures) were imputed by averaging over the same nutrients of the Italian-FCDB-specific related food items (Table S3).

#### 3.2.4. Indirect Matching: Single Dish Ingredients in Nutrition5k Were Composite Recipes

Thirty-five recipes were created for the ingredients in Nutrition5k that were actually composite recipes (35/78~45% of total indirect matching procedures) (Table S4). One example is brownies: the recipe, the ingredients (i.e., chocolate, butter, sugar, flour, eggs, cocoa powder, vanilla extract, and yeast), and their amounts were searched on the previously specified Italian cooking website(s), expressed as a 100 g standard portion, and later adapted to the brownies portion indicated in the Nutrition5k dishes.

### 3.3. Ingredients Portion: Checks

Four ingredients (i.e., *olives, lemon, asparagus,* and *white rice*) were identified as outliers from the summary statistics of the mass distribution across ingredients and dishes. From visual inspection of food images, we discovered that the original portion of a few grams was wrongly reported in the order of magnitude of kilograms (7974 g instead of 7.974 g for olives and lemon; 3324 g instead of 3.324 for asparagus; 2991 g instead of 2.991 g for white rice). We then modified the corresponding mass with the amount reflected in food images.

### 3.4. Handling of Missing Values

After linking each ingredient in Nutrition5k with the corresponding nutritional composition in the Italian FCDB, a total of 2621 missing values were retrieved and needed to be imputed.

Percentages of missing values varied substantially across nutrients, ranging from 0% to 91.1% (vitamin K), with a median of 2.6% (IQR: 0–40.1%), and the second nutrient with the highest percentage of missing values being at 54.7% (i.e., arachidic and beenic acids). High rates of missing values were found in vitamins such as biotin (42.2%), pantothenic acid, and vitamin B12 (both 40.1%). The minerals most affected by missing values were iodine (41.7%), chloride (40.6%), manganese, selenium, cupper, and magnesium (all 40.1%), and sulphur (36.5%). Despite the low missing rate (0–0.5%) in carbohydrates (total, starch, and soluble), minor fractions of carbohydrates showed moderate (galactose: 33.3%) to high (glucose, fructose, lactose, maltose, and sucrose: 40.6%) percentages of missing values. Other macronutrient fractions strongly affected by missing values were polyunsaturated fatty acids (e.g., arachidonic acid: 41.1%, docosaesaenoic acid: 42.2%, and eicosapentenoic acid: 42.7%), monounsaturated fatty acids (e.g., palmitoleic acid: 42.2% and myristoleic acid: 51.0%), and saturated fatty acids (e.g., palmitic acid: 40.1%, and beenic and arachidic acids: 54.7%).

Identified missing values were imputed with the following techniques: values from similar food items (1771, 67.5%), values calculated by difference between nutrients (439, 16.9%), values calculated from recipes (183, 7.1%), presumed values (i.e., logical zero) (140, 5.3%), and values borrowed from other FCDBs (88, 3.4%). In detail, the “values from similar food items” strategy originally allowed 65 values to be imputed, but these were later integrated with 1706 values that had yet to be imputed, while waiting for a future release of the Italian FCDB; this left us with 1771 values in total for this category.

After manual data curation of ingredients, food matching, linkage of nutritional compositions, and imputation of missing values, the dataset was ready for the statistical analyses, including descriptive and inferential statistics procedures.

### 3.5. Distribution of Dish Ingredients by Frequency of Use and Mass

**Figure 3:**
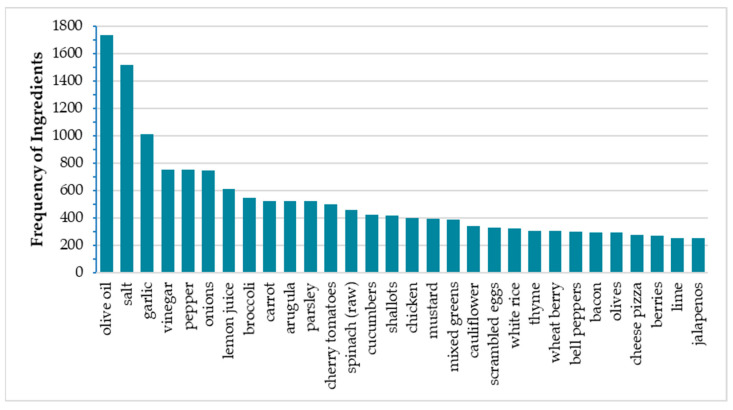
Top 30 ingredients by frequency of use in Nutrition5k after data curation.

Dish complexity in Nutrition5k ranged from a single ingredient to up to 34 ingredients, with a median value of 3 ingredients per dish (IQR: 1–9). The most frequently used ingredients were olive oil, salt, garlic, vinegar, pepper, onions, and lemon juice, with a cumulative frequency of 25% (7127 out of 28,453); in addition to condiments, other frequently used ingredients were broccoli, carrots, arugula, parsley, cherry tomatoes, raw spinach, cucumbers, and shallots, with a cumulative frequency of ~14% (3296 out of 28,401) (Figure 3). The first three condiments (olive oil, salt, and garlic) were used in 20–35% of the 5004 dishes.

**Figure 4:**
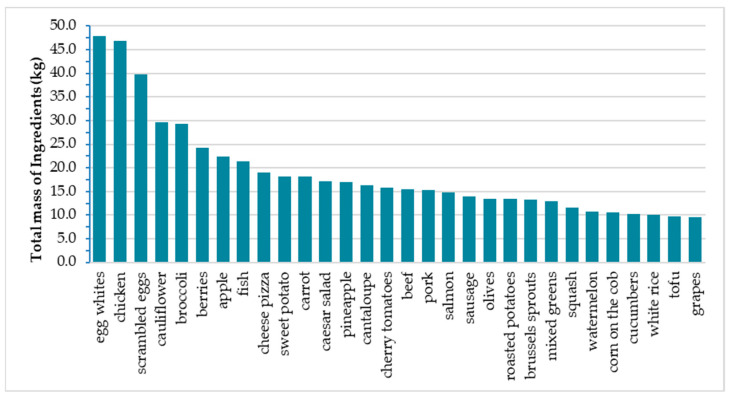
Top 30 ingredients by total mass (kg) across dishes in Nutrition5k after data curation.

To provide a more intuitive overview of the common ingredients used, Figure 4 shows the top 30 ingredients by mass. Instead of condiments, top positions were covered by protein food sources including eggs, white and red meat, fish, and tofu (cumulative frequency of 25%). Fruit (e.g., *apple*, *pineapple*, and *watermelon*) and starchy foods (e.g., *potatoes*, *corn*, and *pizza*) were additionally present in the ranking, whereas vegetables like carrots and broccoli still kept their positions. Compared to the original Nutrition5k dataset [15], olives, lemon, and white rice were downgraded in rank (i.e., olives: from 4th to 20th position, lemon: from 7th to 62nd position, and white rice: from 25th to 28th position), because the material errors on their mass values were corrected.

### 3.6. Distribution of Mass, Energy and Macronutrient Contents across Available Dishes in Nutrition5k after Italian Nutritional Values Were Linked

Table 1 shows descriptive statistics for the mass, energy and macronutrient contents, as derived by attributing Italian-FCDB-specific nutritional values to dishes from Nutrition5k. The minimum value for energy content (1.1 kcal) was given by dish_1551381990 (1 ingredient: asparagus). The minimum value for protein content (0 g) was given by dish_1575997210 (one ingredient: grapes, one unit), which also provided the minimum value for mass (3.0 g). In addition to proteins, the minimum value was also equal to 0 g for fats (99 dishes) and carbohydrates (201 dishes). Distributions of mass and nutrients across dishes were all right-skewed. The median mass was 145 g (IQR: 73–257 g), with a maximum of 1102.0 g (dish_1561739805, 10 ingredients, mostly fruit and vegetables, with scrambled eggs). The median energy content was 162.7 kcal with a maximum of 1488.4 kcal (dish_1567714934, six ingredients; see Table S6). The median total protein content was 8.0 g (IQR: 1.9–21.7 g), with a maximum of 133.7 g (dish_1563305083, 29 ingredients, mostly vegetables and dressings, in addition to animal and vegetable protein sources and cereals). The median total fat content was 7.0 g (IQR: 0.56–17.1 g), with a maximum of 130.7 g (top-energy dish dish_1567714934; see Table S6). The median available carbohydrate content was 9.2 g (IQR: 2.73–20.3 g), with a maximum of 117.7 g (dish_1566329049, 20 ingredients; see Table S9).

### 3.7. Nutrition5k Dataset with Italian versus US Nutritional Values: A Comparison

After having fixed issues described in the Section 2 (Section 2.3 and Section 2.4), we compared the nutrient content (energy, proteins, fats, and carbohydrates) between the Italian and US FCDBs through measures of agreement and differences.

**Figure 5:**
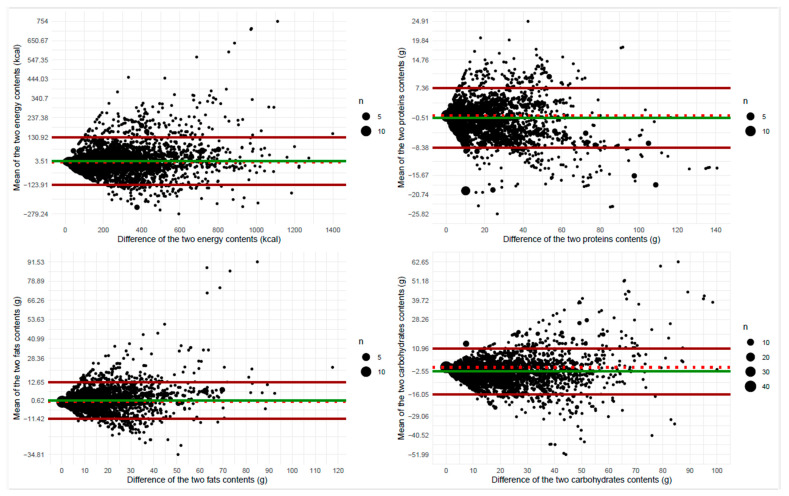
Bland–Altman plots representing the raw absolute difference between Italian- and US-specific content (*x*-axis) versus the mean of the Italian- and US-specific content for each nutrient (*y*-axis), with corresponding 95% limits of agreement (green line for the mean difference and corresponding red lines for the limits of agreement). The dotted red line indicates the reference value of 0.

Figure S6 shows scatterplots of nutritional content obtained across the Italian and US FCDBs, integrated with Pearson correlation coefficients and the corresponding *p*-values from hypothesis testing. Correlation coefficients were equal to ~0.90 for fats and carbohydrates, 0.95 for energy, and 0.98 for proteins (all *p*-values < 0.001), indicating the presence of a strong linear relationship between corresponding nutrients under the Italian and US FCDBs. Table 2 shows percentages of agreement on the classification of dishes into quantiles and corresponding measures of agreement, Cohen’s kappa. Percentages of perfect agreement ranged from 68% (carbohydrates) to 84% (proteins); in the absence of material switching between opposite quintiles, percentages of switching between adjacent quintiles ranged from 16% (proteins) to 30% (carbohydrates). When chance was accounted for, Cohen’s kappa coefficients still ranged from 0.60 (carbohydrates) to 0.80 (proteins), indicating moderate or strong agreement between corresponding nutrients. Figure 5 shows the Bland–Altman plots for each nutrient, with corresponding 95% limits of agreement. The mean difference (i.e., the bias) in nutrient content under the two FCDBs was 3.51 kcal (95% limits of agreement: −123.91, 130.92) for energy, −0.51 g (95% limits of agreement: −8.38, 7.36) for proteins, −0.62 g (95% limits of agreement: −11.42, 12.65) for fats, and −2.55 g (95% limits of agreement: −16.05, 10.96) for carbohydrates. In addition, the variability was not consistent across the plots for all investigated nutrients: the scatter around the bias line was larger as the mean on the *x*-axis became higher.

Figure S7 shows the kernel density estimation plots of the difference in nutritional content obtained across the Italian and US FCDBs. A peak at around 0 (i.e., no difference between nutrient content) was roughly identified for energy, proteins, and fats. Table 3 additionally shows the summary statistics of the dish-specific raw differences: the median values were exactly equal to 0 g for proteins and fats and −0.73 kcal for energy, whereas the median was equal to −2.10 g for carbohydrates (over a 9.2 g median carbohydrate content per dish). Similarly, 75% of the dishes showed, at most, a difference of 15.59 kcal (median energy content per dish: 162.7 kcal; see Table 1), 0.83 g of proteins (median content: 8.0 g), 1.80 g of fats (median content: 7.0 g), and −0.27 g of carbohydrates (median content: 9.2 g). The Kolmogorov–Smirnov test showed deviations from the Normality assumption (all *p*-values < 0.001) for each difference. In the corresponding Wilcoxon signed-rank test, the true location shift was not equal to 0, although the differences in medians were modest (energy: 162.70 vs. 157.60 kcal; proteins: 8.00 vs. 8.39 g; fats: 7.00 vs. 6.72 g; carbohydrates: 9.23 vs. 12.58 g).

Information on the top 25 dishes showing the most extreme differences in absolute value for each investigated nutrient were shown in Tables S6–S9, together with the summary statistics of the differences in absolute value (Table S5). In detail, the maximum values for |ΔEnergy| and |Δfats| both derived from the same dish (dish_1567714934, 6 ingredients), which also showed >3rd quartile values for |Δproteins| and |Δcarbohydrates|. These consistent differences across all macronutrients were mainly due to a different nutritional composition of Caesar dressing, a newly created recipe with a full-fat content in the Italian-FCDB-specific nutritional composition for the mixed salad dish. The maximum value for |Δproteins| was provided by dish_1558115364 (four ingredients: potatoes, almonds, bacon, and a whole apple); this dish also presented values for |Δenergy| > 80th percentile, |Δfats| > 70th percentile, and |Δcarbohydrates| > 85th percentile. These consistent differences across all macronutrients were mainly due to a different nutritional composition of bacon, which is available in the Italian FCDB only as a raw food item. The maximum value for |Δcarbohydrates| was given by dish_1561492228 (24 ingredients: mostly vegetables, but with 2 protein sources and 3 cereals, (i.e., millet, fried rice, and wheat berry)), which also provided |Δenergy| > 95th percentile and |Δfats| > 80th percentile, but |Δproteins| < 20th percentile. Identified differences were still due to the absence of cooked millet in the Italian FCDB and the creation of the corresponding recipe for fried rice with the raw cereal ingredients, instead of the cooked ones.

Table 4 shows results from the multiple robust regression models including each nutrient’s difference as the dependent variable and independent variables given by: dish mass, number of ingredients per dish, presence of recreated recipes in each dish, and differential use of raw and cooked ingredients in each dish, as well as the interaction term between differential use of raw and cooked ingredients and presence of recreated recipes in each dish.

For energy content, as compared to “no difference in raw and cooked ingredients/no recreated recipes”, the joint presence of recreated recipes and differences in raw and cooked ingredients across the Italian and US FCDBs was related to an increased nutrient difference (*p*-value from robust ANOVA < 0.001): the beta coefficients reached 18.99 (95% CI: 14.85, 23.14) for “one difference in raw and cooked ingredients/recreated recipes” and 16.73 (95% CI: 12.67, 20.79) for “more than one difference/recreated recipes”. The main effects for total mass and number of ingredients were, however, not materially related to the difference in energy content.

For the carbohydrate content, each 25 g increment in total mass was related to a decreased difference in nutrients under the two FCDBs (beta coefficient: −0.37, 95% CI: −0.38, −0.35); each additional ingredient was related to an increased difference in the nutrient under the two FCDBs (beta coefficient: 0.11, 95% CI: 0.10, 0.12), and differences in raw and cooked ingredients were related to an increased difference in the nutrient (beta coefficient, 1 ingredient difference: 0.51, 95% CI: 0.39, 0.62; beta coefficient, >1 ingredients difference: 1.54, 95% CI: 1.38, 1.69, *p*-value from robust ANOVA < 0.001); the main effects for recreated recipes (no/yes) were materially unrelated to the difference in carbohydrate content.

As compared to carbohydrates, fats and proteins showed opposite effects for total mass (proteins: beta coefficient = 0.10, 95% CI: 0.10, 0.11; fats: beta coefficient = 0.10, 95% CI: 0.10, 0.11) and number of ingredients (proteins: beta coefficient = −0.15, 95% CI: −0.16, −0.15; fats: beta coefficient = −0.04, 95% CI: −0.04, −0.03). In addition, as compared to “no difference in raw and cooked ingredients/no recreated recipes”, the joint presence of recreated recipes and differences in raw and cooked ingredients across the Italian and US FCDBs was significantly related to the corresponding nutrient difference (proteins: *p*-value from robust ANOVA = 0.002; fats: *p*-value from robust ANOVA < 0.001). The beta coefficients for fats reached 1.04 (95% CI: 0.71, 1.37) for “one difference in raw and cooked ingredients/recreated recipes” and 1.88 (95% CI: 1.56, 2.21) for “more than one difference/recreated recipes”; while significant with “one difference/recreated recipes” (beta coefficient = 0.60, 95% CI: 0.39, 0.81), the beta coefficient for the proteins content lost significance in the extreme category.

## 4. Discussion

In order to develop and train a novel Italian machine-learning-oriented food-image-based dietary assessment tool, a critical step is to link dish images available from other countries with nutritional values of Italian foods and recipes.

To our knowledge, no study so far has provided this cross-country harmonization process of FCDBs within an image-based machine learning framework. The harmonization process involved several steps, including (1) manual data curation of dishes and dish ingredients in Nutrition5k; (2) exact and indirect food matching between ingredients in Nutrition5k and food items in the Italian FCDB; (3) creation of 35 recipes; (4) identifying 67 discrepancies, including 21 in recipes, in the use of raw and cooked ingredients across FCDBs; and (5) handling of 2621 missing values. It finally allowed about 90 nutrients to be “linked” to the original dishes, enabling further statistical analyses to be performed. Overall, the 5004 selected dishes from Nutrition5k had a 145 g median mass and a median number of 3 ingredients. Measures of agreement and differences [32] of energy, proteins, fats, and carbohydrates—the only nutrients available from Nutrition5k—across the Italian and US FCDBs generally support modest differences between corresponding nutrients estimated under the two FCDBs. However, the four Bland–Altman plots additionally showed that the variability was not consistent. This phenomenon was similarly observed in a previous publication [32] comparing macro- and micro-nutrient intakes from the USDA and the European Prospective Investigation into Cancer and Nutrition (EPIC).

Among the three macronutrients, carbohydrates showed the minimum values for Pearson correlation coefficients, percentages of agreement, and Cohen’s kappa; in addition, the mean difference in the Bland–Altman plot was the highest in absolute value, as compared to the distribution range (i.e., −2.55 over about 114 g). Similarly, descriptive statistics suggested a median difference for carbohydrates of −2.10 g, compared to 0 for proteins and fats. This means that the Italian FCDB generally provided smaller values for the carbohydrate content, as compared to the US FCDB. This is explained in part by a different definition of carbohydrates across the two FCDBs, which reflects more general differences in definition and methods used (analytical or calculations) for carbohydrates across FCDBs [2,32]. In detail, within the US FCDB, the total carbohydrate content is calculated ‘by difference’ (i.e., the difference between 100 and the sum of the percentages of water, proteins, total fats, ash and, when present, alcohol), and therefore includes total dietary fiber [16]. By contrast, in the Italian FCDB, “available carbohydrates” is defined as the sum of soluble carbohydrates and starch, and it thus does not include fiber [21]. It is, therefore, highly expected that the Italian FCDB underestimates in mean/median carbohydrates, as compared to the US FCDB.

Other possible sources of discrepancies between corresponding nutrients under the Italian and US FCDBs were suggested to us by a descriptive analysis of the top 25 dishes showing the most extreme (i.e., >99.5%) differences in nutrient absolute values across the two FCDBs. In detail, we identified recreated recipes (e.g., *Caesar dressing*/*salad*, *cheese pizza*, *scrambles eggs*, *fried rice*, and *vinaigrette*) for most of the top dishes. Across them, we also identified bacon, salmon, tuna, millet, and beef, which suggested a discrepancy in the use of raw and cooked food items across the two FCDBs. Finally, the top 25 dishes also showed a consistently high median number of ingredients, potentially due to the presence of recipes. Based on this exploration, we hypothesized that the role of the following potential determinants should be formally investigated: (1) dish mass; (2) the number of ingredients in each dish; (3) the presence of one or more recreated recipes per dish; and (4) the differential use of raw and cooked ingredients in each dish. We analyzed their impact on each nutrient’s difference using multiple regression models. Results from the regression models were reassuring in that they confirmed that each of the previous determinants had a role in explaining potential differences across the available FCDBs. In addition, the statistical significance observed for the only interaction term inserted into the models (presence of recipes and differential use of raw and cooked ingredients) further supported a combined role of some of these determinants. Although expected, this aspect should be better investigated in future projects with larger datasets to confirm the direction and strength of the relationship for each investigated nutrient.

This work presents both strengths and limitations. In the absence of a recognized gold standard or a validated procedure, we developed the entire harmonization process, including food matching between the Nutrition5k dishes and the Italian FCDB, by following previous studies [2,17,32] and suggestions from EUROFIR [41] and the Food and Agriculture Organization of the United Nations [26] evaluated on the basis of senior nutritionists’ experience. Within this general process, we dedicated special care to improve on the automatic process followed in Nutrition5k (i.e., incremental weighing, scanning, and logging of each item, while added to the plate in the Google cafeterias immediately before consumption [15]). In detail, we exploited dish photos to: (1) check the presence of “plate only” dishes; (2) regain missing-name ingredients, check their mass as originally provided in Nutrition5k, and add their corresponding nutritional values; (3) correct outliers in terms of mass; and (4) identify generic ingredients and recipes for some of the indirect matching procedures. Overall, the comparison analysis revealed a good level of agreement for energy and macronutrients, despite us introducing a certain level of “disagreement” in the harmonization process (e.g., by creating new recipes). Third, compared to the existing literature (e.g., [32]), we additionally built multiple regression models to investigate potential determinants of differences between Italian- and US-FCDB-specific nutritional values for the few nutrients available in Nutrition5k.

A first limitation of this project is related to the country-specific nature of dishes from Nutrition5k. For example, the Italian FCDB did not include some varieties of fruits and vegetables from Asian or Central American countries. Although this issue impacted only a modest proportion of ingredients, indirect matching led to the creation of new recipes, which may have contributed to increased differences in nutritional values between the two databases. Second, we recognized that some cooked ingredients in Nutrition5k could only be matched with raw food items in the Italian FCDB. This issue may have particularly affected the carbohydrate content of dishes; indeed, when cooked, dishes rich in starch increase in weight, whereas those rich in proteins and fats lose weight (e.g., 100 g of raw pasta = 188 g of boiled pasta, yield factor = 1.9; 100 g of raw steak = 74 g of roasted steak, yield factor = 0.7 [42]). Third, we could not account for yield factors either in food matching or in the creation of new recipes; indeed, most ingredients in the Italian FCDB lack yield factors or, as in recipe creation, we lacked ancillary information (e.g., cooking method and time) needed to apply the yield factors. Fourth, the comparison of the nutrient content between the Italian and US FCDBs was limited to energy and macronutrients, because Nutrition5k lacks information on micronutrients. However, as highlighted in previous publications [2,32], micronutrients are less likely to be comparable due to intrinsic compilation differences across FCDBs. Finally, the presence of missing values, although reduced at the minimum by expert nutritionist manual imputation, is a limitation common to all FCDBs in general.

## 5. Conclusions

To the best of our knowledge, this article is the first to explore and provide solutions to all of the methodological issues encountered when adapting the nutritional composition of foods from one country to food images collected in another country. These food images will be used as the input for a machine-learning application for dietary assessment. Compared to the automatic process followed in Nutrition5k, our findings confirmed the importance of manual data curation performed by expert nutritionists at any step of the harmonization procedure. In the comparison between energy and macronutrient contents obtained from the Italian and US FCDBs across dishes, the agreement was consistently moderate or even satisfactory across the different statistical approaches followed. To our knowledge, we are the first to further investigate potential determinants of differences in energy and macronutrients, identifying a role for each determinant alone and/or in combination with others. However, further efforts are needed to confirm the strength and direction of the associations investigated in other image databases and spot other potential determinants of differences. Further cross-country efforts in harmonization of standards are needed to improve data quality, availability, and reliability in the era of machine-learning-oriented dietary assessment. In the framework of precision medicine, the holistic integration of dish images with omics profiles within the same data collection tool would allow more comprehensive monitoring of health status [43] and putative risk factors [44].

## Figures and Tables

**Table 1:** Descriptive statistics for mass, energy and macronutrient contents of dishes after attributing Italian-FCDB-specific nutritional values to dishes from Nutrition5k.

	Mass (g)	Energy (kcal) ^1^	Proteins (g)	Fats (g)	Carbohydrates (g)
Minimum	3.0	1.1	0.0	0.0	0.0
First quartile	73.0	62.5	1.9	0.6	2.7
Median	145.0	162.7	8.0	7.0	9.2
Third quartile	257.0	324.7	21.7	17.1	20.3
Maximun	1102.0	1488.4	133.7	130.7	117.7
Mean	182.7	220.8	14.5	11.5	14.2
SD	143.1	205.7	17.3	13.9	15.5

^1^ In the Italian FCDB, the energy content of each dish included fiber; therefore, for each dish, the sum of the calories provided by total proteins, total fats, and available carbohydrates was not equal to the total energy.

**Table 2:** Classification of dishes into quintiles and Cohen’s kappa coefficient under the Italian and the US FCDBs.

**Energy (kcal)**							**Proteins (g)**						
		US FCDB							US FCDB				
Italian FCDB		Q1	Q2	Q3	Q4	Q5	Italian FCDB		Q1	Q2	Q3	Q4	Q5
	Q1	18.17	1.84	0.0	0.0	0.0		Q1	18.35	1.44	0.0	0.22	0.0
	Q2	1.56	15.61	2.84	0.0	0.0		Q2	1.66	16.15	2.22	0.02	0.0
	Q3	0.28	2.26	14.13	3.30	0.02		Q3	0.0	2.30	15.47	2.14	0.04
	Q4	0.0	0.26	2.80	13.63	3.32		Q4	0.0	0.14	2.26	15.67	1.94
	Q5	0.0	0.06	0.22	3.06	16.67		Q5	0.0	0.0	0.02	1.96	18.03
					kappa: 0.73							kappa: 0.80	
**Fats (g)**							**Carbohydrates (g)**						
		US FCDB							US FCDB				
Italian FCDB		Q1	Q2	Q3	Q4	Q5	Italian FCDB		Q1	Q2	Q3	Q4	Q5
	Q1	17.81	2.20	0.0	0.0	0.0		Q1	16.89	3.02	0.12	0.0	0.0
	Q2	2.28	13.97	3.56	0.20	0.0		Q2	2.70	12.17	4.40	0.70	0.02
	Q3	0.0	2.94	12.19	4.76	0.10		Q3	0.20	4.50	11.07	4.00	0.22
	Q4	0.0	0.64	3.76	11.15	4.46		Q4	0.22	0.36	4.08	11.75	3.62
	Q5	0.0	0.18	0.48	3.90	15.45		Q5	0.0	0.0	0.3	3.56	16.13
					kappa: 0.63							kappa: 0.60	

Abbreviations: FCDB, Food Composition Database.

**Table 3:** Summary statistics of raw differences (i.e., nutrients in the Italian FCDB–nutrients in the US FCDB) in the content of energy and macronutrients across Italian-FCDB-specific and US-FCDB-specific values.

	Energy Difference (kcal)	Proteins Difference (g)	Fats Difference (g)	Carbohydrates Difference (g)
Minimum	−279.24	−25.82	−34.81	−52.00
First quartile	−18.08	−1.46	−0.73	−5.08
Median	−0.73	0.0	0.0	−2.10
Third quartile	15.59	0.83	1.80	−0.27
Maximun	754.0	24.91	91.53	62.65
Mean	3.50	−0.51	0.62	−2.54
SD	65.01	4.02	6.14	6.89

Abbreviations: FCDB, Food Composition Database.

**Table 4:** Beta coefficients and corresponding 95% confidence intervals (95% CI) from the multiple robust regression models ^1^.

		**Difference of Energy** **Content (kcal)**			**Difference of Proteins** **Content (g)**			**Difference of Fats** **Content (g)**			**Difference of Carbohydrates** **Content (g)**		
**Characteristic**	**N**	**Beta**	**95% CI**	***p*-Value**	**Beta**	**95% CI**	***p*-Value**	**Beta**	**95% CI**	***p*-Value**	**Beta**	**95% CI**	***p*-Value**
**Intercept**	5004	−0.48	−1.15, 0.19	0.550 ^2^	−0.16	−0.19, −0.12	**<0.001** ^2^	0.03	−0.02, 0.09	0.600 ^2^	−1.50	−1.59, −1.42	**<0.001**
**Total mass (25 g increase)**	5004	0.02	−0.06, 0.10	0.811 ^2^	0.10	0.10, 0.11	**<0.001** ^2^	0.10	0.10, 0.11	**<0.001** ^2^	−0.37	−0.38, −0.35	**<0.001** ^2^
**Number of ingredients**	5004	0.00	−0.09, 0.08	0.969 ^2^	−0.15	−0.16, −0.15	**<0.001** ^2^	−0.04	−0.04, −0.03	**<0.001** ^2^	0.11	0.10, 0.12	**<0.001** ^2^
**Differential use of raw/cooked ingredients**				**<0.001** ^3^			**<0.001** ^3^			**<0.001** ^3^			**<0.001** ^3^
*No difference in ingredients*	1768	—	—		—	—		—	—		—	—	
*One ingredient difference*	1500	−2.87	−3.81, −1.93	**0.011** ^2^	0.13	0.08, 0.18	**0.036** ^2^	−0.53	−0.61, −0.46	**<0.001** ^2^	0.51	0.39, 0.62	**<0.001** ^2^
*More ingredients difference*	1736	−10.87	−12.17, −9.57	**<0.001** ^2^	−0.21	−0.28, −0.13	**0.020** ^2^	−1.83	−1.94, −1.73	**<0.001** ^2^	1.54	1.38, 1.69	**<0.001** ^2^
**Recreated recipes in dish**				**<0.001** ^3^			**<0.001** ^3^			**<0.001** ^3^			0.351 ^3^
*No*	3661	—	—		—	—		—	—		—	—	
*Yes*	1343	−4.06	−7.89, −0.23	0.375 ^2^	0.66	0.46, 0.85	**0.005** ^2^	0.71	0.41, 1.02	**0.050** ^2^	0.52	0.17, 0.88	0.217 ^2^
**Interaction between differential use of raw/cooked ingredients and recreated recipes**	5004			**<0.001** ^3^			**0. 002** ^3^			**<0.001** ^3^			0.252 ^3^
*One difference: Yes*	399	18.99	14.85, 23.14	**<0.001** ^2^	0.60	0.39, 0.81	**0. 018** ^2^	1.04	0.71, 1.37	**0.008** ^2^	−0.49	−0.89, −0.09	0.304 ^2^
*More differences: Yes*	863	16.73	12.67, 20.79	**0.001** ^2^	0.42	0.21, 0.63	0.098 ^2^	1.88	1.56, 2.21	**<0.001** ^2^	−0.69	−1.07, −0.30	0.136 ^2^

^1^ Estimates were obtained by including each nutrient’s difference as the dependent variable and dish mass, number of ingredients per dish, presence of recreated recipes in each dish, and differential use of raw and cooked ingredients in each dish, as well as the interaction term between differential use of raw and cooked ingredients and presence of recreated recipes in each dish. *p*-values in bold typeface were those <0.05. ^2^ This *p*-value was derived from the robust tests for the single level of each variable. ^3^ This *p*-value was derived from the robust ANOVA tests for the single variable.

## Data Availability

The current analysis was based on a publicly archived dataset available at https://github.com/google-research-datasets/Nutrition5k accessed on 15 July 2024. Codes used to perform the described statistical analysis are available upon request from the corresponding author via email.

## Supplementary Materials

The following supporting information can be downloaded at https://www.mdpi.com/article/10.3390/nu16193339/s1: Table S1: Indirect matching: ingredients from Nutrition5k matched with the most similar food items in the Italian FCDB; Table S2: Indirect matching: ingredients from Nutrition5k unavailable in the Italian FCDB and matched with the US FCDB; Table S3: Indirect matching: generic ingredients from Nutrition5k were imputed with a group of related food items from the Italian FCDB, whose nutrients were averaged; Table S4: Indirect matching: composite recipes expressed as ingredients in Nutrition5k were created by using Italian FCDB food items; Table S5: Summary statistics of differences (absolute value) in energy and macronutrient content of the Nutrition5k dishes across Italian-FCDB-specific and US-FCDB-specific values; Table S6: Top 25 dishes showing the most extreme (i.e., >99.5%) differences in absolute value across FCDBs for energy content; Table S7: Top 25 dishes showing the most extreme (i.e., >99.5%) differences in absolute value across FCDBs for protein content; Table S8: Top 25 dishes showing the most extreme (i.e., >99.5%) differences in absolute value across FCDBs for fat content; Table S9: Top 25 dishes showing the most extreme (i.e., >99.5%) differences in absolute value across FCDBs for carbohydrate content; Figure S1: Nutrition5k dataset in the long format, with each ingredient matched with the corresponding food item from the Italian FCDB and the corresponding Italian-FCDB-specific nutritional composition; Figure S2: Imputation of ingredients from Nutrition5k by similarity: frequency of specific ingredients; Figure S3: Imputation of ingredients from Nutrition5k by international sources: frequency of specific ingredients; Figure S4: Imputation of ingredients from Nutrition5k by group of related food items: frequency of specific ingredients; Figure S5: Imputation of ingredients from Nutrition5k by creation of composite recipes: frequency of specific ingredients; Figure S6: Scatterplots of nutritional content obtained across the Italian and US FCDBs; Figure S7: Kernel density estimation plots of the difference between the Italian and US FCDBs for energy and macronutrients.
