# Supplementary material for: Tailoring the Nutritional Composition of Italian Foods to the US Nutrition5k Dataset for Food Image Recognition: Challenges and a Comparative Analysis
Source: Nutrients. 2024 Oct 1;16(19):3339. doi: 10.3390/nu16193339 (PMC11479105; doi:10.3390/nu16193339)
Supplement: Supplementary file 1 [file nutrients-16-03339-s001.zip › nutrients-3162461-supplementary.pdf]

Table S1. Indirect matching: ingredients from Nutrition5k matched with the most similar food items in Italian FCDB.

Ingredients from the Nutrition5k dataset	Food items from the Italian FCDB
Bagels	BREAD, prepared with milk
Black beans	BEANS, canned
Blue cheese	GORGONZOLA CHEESE
Bok choy	SWISS CHARD
Buttermilk	MILK, COW, SKIMMED, PASTEURIZED
Chayote squash	PUMPKIN or SQUASH
Country rice	RICE, BROWN, WHOLEGRAIN, cooked
Cranberries	RAISINS
Green onions	SHALLOTS
Hominy	SWEETCORN, CANNED, drained
Jalapenos	PEPPERS, CHILI, RED
Jicama	POTATOES
Kale	CABBAGE, JANUARY KING
Noodles	PASTA, WHITE, dried, cooked
Nopales	PRICKLY PEAR
Parsnips	CARROTS
Pepperoni	SALAMI, pork and beef meat
Pilaf	RICE SALAD (prepared from recipe)
Pinto beans	BEANS, canned
Rice noodles	PASTA, WHITE, dried, cooked
Snow peas	PEAS, canned
Tacos	PIADINA (Italian flatbread)
Tatsoi	SWISS CHARD
Tomatillo	TOMATOES
Turkey bacon	BACON (pork meat, salted, smoked)
Wild rice	RICE, VENERE, cooked
Yam	BATATAS or SWEET POTATOES

Abbreviations: FCDB, Food Composition Database.

Table S2. Indirect matching: ingredients from Nutrition5k unavailable in the Italian FCDB and matched with US FCDB.

**Ingredients from the Nutrition5k dataset**	**Food items from the US FCDB**
Barbecue sauce	Sauce, barbecue
Chia seeds	Seeds, chia seeds, dried
Cilantro	Coriander (cilantro) leaves, raw
Edamame	Edamame, frozen, unprepared
Ginger	Ginger root, raw
Ketchup	Catsup
Mustard	Mustard
Pumpkin seeds	Seeds, pumpkin and squash seed kernels, dried
Sunflower seeds	Seeds, sunflower seed kernels, dried
Teriyaki sauce	Sauce, teriyaki, ready-to-serve

Abbreviations: FCDB, Food Composition Database.

Table S3. Indirect matching: generic ingredients from Nutrition5k were imputed with a group of related food items from the Italian FCDB, whose nutrients were averaged.

Ingredients from the Nutrition5k dataset	Italian FCDB food items used for mean value calculation
Berries	Mulberries; Blackberries; Strawberries; Raspberries; Blueberries or bilberries
Mushroom	Mushrooms, "Ovuli" TYPE; Mushrooms, "Porcino" TYPE; Mushrooms, common; Mushrooms, "Porcinelli" TYPE; Mushrooms, Honey; Mushrooms, Morel; Mushrooms, Chanterelle
Cheese, and *Goat cheese*	Asiago cheese; Bra cheese; Crescenza cheese; Edam cheese, aged; Cottage cheese; Gouda cheese, fresh; Gouda cheese, aged; Montasio cheese; Pecorino cheese, for grating; Pecorino romano cheese; Provoloncino cheese, sweet; Provolone cheese, hot; Ricotta cheese, german type, whole; Ricotta cheese, german type, light; Robiola cheese; Taleggio cheese; Provola cheese, smoked; Ricotta cheese (made from cow milk); Mozzarella cheese (buffalo); Soft cheese (Bel Paese type); Burrini cheese; Caciocavallo cheese; Caciotta romana cheese (made with sheep milk); Caciotta toscana cheese; Caciottina cheese, fresh; Cheddar cheese; Dolce Verde cheese; Emmental cheese; Fior di latte cheese; Fontina cheese; processed cheese; processed cheese, low fat; Gorgonzola cheese; Grana cheese; Groviera cheese; Latteria cheese; Mascarpone cheese; Mozzarella cheese (cow); Pastorella cheese; Pecorino cheese; Provolone cheese; Ricotta cheese (made from sheep milk); Scamorza cheese; Stracchino cheese; Parmigiano cheese; Processed cheese, plain, slices; Full fat soft cheese (Philadelphia type); Pecorino cheese, fresh; Rochefort cheese; Edam cheese, fresh; Caciocavallo cheese, smoked; Caciotta cheese, smoked; Scamorza cheese, smoked; Camembert cheese; Brie cheese; Feta cheese; soft cheese, light; Butirro calabro cheese; Cacioricotta cheese; Caciottina cheese, made with sheep and cow milk; Italico cheese; Pecorino siciliano cheese; Ricotta cheese (made from buffalo milk); Caciotta cheese, made with sheep and cow milk; Caciottina cheese, made with cow milk; Gorgonzola and Mascarpone (mixed cheese); Gorgonzola cheese with nuts; Burrata cheese; Ricotta cheese (made from cow and sheep milk)
Nuts	Peanuts, raw; Peanuts, roasted; Walnuts, fresh; Walnuts, dried; Hazelnuts, dried; Pine nuts; Pistachio nuts, fresh; Pistachio nuts, dried; Pistachio nuts, roasted and salted; Peanuts, roasted and salted; Pecan nuts; Cashew nuts; Pistachio nuts, *"Pistacchio di Bronte PDO"*
Seeds	Fennel, seeds; Cumin seeds; Linseed; Herbs (seeds); Seeds, pumpkin and squash seed kernels, dried[1]; Seeds, chia seeds, dried[1], Seeds, sunflower seed kernels, dried[1]

[1]For the entry "seeds", the Italian FCDB food items were integrated with previously selected US FCDB food items to improve comparability across indirect matching procedures. Abbreviations: FCDB, Food Composition Database.

Table S4. Indirect matching: composite recipes expressed as ingredients in Nutrition5k were created by using Italian FCDB food items.

Ingredients from the Nutrition5k dataset	Italian FCDB food items used to create recipes
Brownies[a]	Chocolate, dark (cocoa 70%); Butter; Sugar, cane, brown; Flour, wheat, type 00 (fine ground); Eggs, chicken, whole; Cocoa, unsweetened, powder; Vanilla extract, with alcohol; Yeast, powder
Caesar dressing[a]	Vegetable oil, olive, extravirgin; Juice, lemon, fresh; Lemon, peel; Eggs, chicken, yolk; Mustard[1]; Sauce, worcestershire[1]
Caesar salad[a]	Lettuce; Parmigiano cheese; Croutons; Vegetable oil, olive, extravirgin; Juice, lemon, fresh; Lemon, peel; Eggs, chicken, yolk; Mustard[1]; Sauce, Worcestershire[1]; Chicken breast, without skin, cooked, pan fried
Cheese pizza[b]	Pizza with mozzarella and tomatoes; Mozzarella cheese, cow; Gorgonzola cheese; Scamorza cheese; Taleggio cheese
Chicken salad[a]	Lettuce; Parmigiano cheese; Vegetable oil, olive, extravirgin; Juice, lemon, fresh; Lemon, peel; Eggs, chicken, yolk; Mustard[1]; Sauce, Worcestershire[1]; Chicken breast, without skin, cooked, pan fried
Chilaquies[a]	Tortillas; Peppers, chili, red; Onions; Tomatoes; Garlic, fresh; Vegetable oil, olive, extravirgin; Eggs, chicken, whole; Avocado
Chili[a]	Beef, 15-18 months, fat and lean meat, with visible fat; Peppers, sweet type; Peppers, chili, red; Onions; Cumin, seeds; Sugar, cane, brown; Beans, canned; Tomato puree' (dry matter 18%); Garlic, fresh; Vegetable oil, olive, extravirgin; Coriander (cilantro) leaves, raw; Pepper, black
Coleslaw[a]	Cabbage, january king; Onions; Carrots; Yoghurt, whole milk; Mustard[1]; Chives, fresh; Vinegar; Sugar, white; Mayonnaise
Crepes[a]	Butter; Flour, wheat, type 00 (fine ground); Eggs, chicken, whole; Milk, cow, semi-skimmed, pasteurized
Curries[a]	Chicken, leg-lower part, without skin; Spices, curry powder[1]; Coriander (cilantro) leaves, raw[1]; Onions; Vegetable oil, olive, extravirgin; Cream (20% fat); Cinnamon; Pepper, black
Dumplings[a]	Flour, wheat, type 00 (fine ground); Water; Sausages, pork meat, raw; Swiss chard (bok choy); Vegetable oil, sesame; Tamari, soya sauce
Fried rice[b]	Rice, white, polished, raw; Vegetable oil, olive, extravirgin
Frozen yogurt[a]	Yoghurt, whole milk; Cream (20% fat); Sugar, white; Chocolate, milk
Greek salad[b]	Tomatoes; Cucumber; Feta cheese; Olives, black type; Vegetable oil, olive, extravirgin
Ground turkey[b]	Turkey hen, whole, without skin; Eggs, chicken, whole; Grana cheese; Parsley, fresh; Vegetable oil, olive, extravirgin
Hash browns[a]	Potatoes; Starch, potato; Onions; Vegetable oil, mixed seeds
Hummus[a]	Chick peas, canned; Tahina (seeds, sesame butter, tahini, from roasted and toasted kernels (most common type))[1]; Vegetable oil, olive, extravirgin; Juice, lemon, fresh
Kimchi[a]	Cabbage, january king; Onions; Carrots; Daikon; Ginger root, raw; Chives, fresh; Garlic, fresh; Sugar, white; Flour, rice; Water
Lasagna[b]	Pasta, made with eggs, dried, raw; Beef, 15-18 months, fat and lean meat, with visible fat; Onions; Carrots; Celery; Tomatoes, canned, whole content; Milk, cow, semi-skimmed, pasteurized; Grana cheese; Flour, wheat, type 00 (fine ground); Vegetable oil, olive, extravirgin; Butter
Mashed potatoes[b]	Potatoes; Milk, cow, whole, pasteurized; Butter
Oatmeal[a]	Flakes, oat; Milk, cow, semi-skimmed, pasteurized; Honey
Omelette[b]	Eggs, chicken, whole; Vegetable oil, olive, extravirgin

Table S4 (Cont'd)

Pasta salad[c]	Salad, unspecified; Pasta, white, dried, raw
Pepperoni pizza[b]	Pizza with mozzarella and tomatoes; Salami, pork and beef meat
Pesto[b]	Pine nuts; Pecorino cheese, for grating; Vegetable oil, olive, extravirgin; Garlic, fresh; Basil, fresh
Roasted potatoes[b]	Potatoes; Vegetable oil, olive, extravirgin
Sandwiches, and *toast*[b]	Bread, white, sandwich type, packaged; Ham; Processed cheese, plain, slices
Scrambled eggs[b]	Eggs, chicken, whole; Vegetable oil, olive, extravirgin
Sour cream[a]	Cream (20% fat); Yoghurt, whole milk
Succotash[a]	Sweetcorn, canned, drained; Beans, canned; Tomatoes; Onions; Vegetable oil, olive, extravirgin
Tortillas[a]	Flour, corn; Vegetable oil, unspecified; Water
Tuna salad[c]	Tuna, canned in oil, drained; Onions; Carrots; Celery
Vinaigrette[a]	Vegetable oil, olive, extravirgin; Vinegar
Waffles[a]	Butter; Sugar, white; Flour, wheat, type 00 (fine ground); Eggs, chicken, whole; Vanilla extract, with alcohol; Yeast, powder

[1]For the indicated ingredient in the recipe, the corresponding food item was sourced from the selected US FCDB. The recipe composition was obtained from the following sources in order of appearance of the corresponding ingredients: dedicated websites for recipes (a) [28, 29], food atlas (b) [27], food image from Nutrition5k dataset (c) [15]. Abbreviations: FCDB, Food Composition Database.

Table S5. Summary statistics of differences (absolute value) in energy and macronutrient content of the Nutrition5k dishes across Italian FCDB-specific and US FCDB-specific values.

	**∣ΔEnergy∣ (kcal)**	**∣ΔProteins∣ (g)**	**∣ΔFats∣ (g)**	**∣ΔCarbohydrates∣ (g)**
**0th percentile (Minimum)**	0.00	0.00	0.00	0.00
5th percentile	0.80	0.01	0.00	0.19
10 th percentile	1.55	0.07	0.03	0.39
15 th percentile	2.61	0.12	0.06	0.59
20 th percentile	3.71	0.18	0.09	0.90
**25 th percentile (1st quartile)**	5.10	0.25	0.14	1.21
30 th percentile	6.80	0.33	0.20	1.52
35 th percentile	8.58	0.44	0.31	1.85
40 th percentile	11.09	0.58	0.53	2.18
45 th percentile	13.83	0.80	0.86	2.53
**50 th percentile (Median)**	17.01	1.03	1.26	2.87
55 th percentile	20.52	1.31	1.65	3.33
60 th percentile	24.78	1.65	2.15	3.88
65 th percentile	29.18	2.10	2.78	4.50
70 th percentile	35.47	2.57	3.59	5.27
**75 th percentile (3rd quartile)**	42.97	3.20	4.33	6.19
80 th percentile	52.98	4.03	5.53	7.20
85 th percentile	68.92	5.10	6.98	8.68
90 th percentile	94.29	6.51	8.67	11.03
95 th percentile	133.99	8.80	12.10	14.60
**100 th percentile (Maximum)**	754.00	25.82	91.53	62.65
**Mean**	35.85	2.37	3.20	4.70
**SD**	54.34	3.28	5.28	5.65

Abbreviations: FCDB, Food Composition Database; SD, standard deviation; USDA, United States Department of Agriculture.

Table S6. Top 25 dishes showing the most extreme (i.e., >99.5%) differences in absolute value across FCDBs for energy content.

DISH_ID	INGREDIENTS	ǀΔ Energyǀ (kcal)	ǀΔ Proteinsǀ (g)	ǀΔ Fatsǀ (g)	ǀΔ Carbohydratesǀ (g)
dish_1567714934	Parmesan cheese, **Caesar dressing**[1], avocado, mixed greens, chicken, olives	754.00 (ITA)[2]	8.32	91.53	15.35
dish_1566850031	Olives, carrot, chicken, **Caesar dressing**, cherry tomatoes, onions	715.80 (ITA)	9.06	87.68	11.37
dish_1566246626	Feta cheese, *broccoli*[1], mixed greens, chicken, olives, **Caesar dressing**	710.47 (ITA)	5.77	85.51	13.51
dish_1566332268	Olives, chicken, spinach (raw), avocado, **Caesar dressing**, cucumbers	637.40 (ITA)	3.94	74.52	14.61
dish_1565987201	Cucumbers, spinach (raw), parmesan cheese, **Caesar dressing**, chicken, olives	589.94 (ITA)	7.23	71.08	10.04
dish_1565123881	**Caesar salad**, olive oil, onions, **cheese pizza**, *zucchini*, salt, spinach (cooked), *millet*, *beef*, *shallots*, *bok choy*, *chayote squash*, vinegar	562.49 (ITA)	24.91	44.72	12.27
dish_1566502211	**Caesar salad**	453.86 (ITA)	20.56	36.11	8.65
dish_1566502271	*Chard*, parsley, *chive*, olive oil, carrot, **Caesar salad**, garlic, salt, basil, chicken, lemon juice, brown rice, rosemary	449.37 (ITA)	17.56	37.82	8.54
dish_1561492228	Cherry tomatoes, *fish*, blueberries, *jalapenos*, vinegar, pepper, salt chicken, parsley, lemon juice, tomatoes, garlic mustard, olive oil, *chive*, *shallots*, spinach (raw), *chard*, radishes, *millet*, bell peppers, **fried rice**, onions, wheat berry	390.08 (ITA)	0.16	5.68	62.65

Table S6 (Cont'd)

dish_1565725244	Parmesan cheese, cherry tomatoes, chicken, bell peppers, carrot, olives, onions, **Caesar dressing**	380.81 (ITA)	8.35	50.59	13.03
dish_1567801468	**Caesar salad**	375.34 (ITA)	17.01	29.86	7.15
dish_1565204931	Onions, vinegar, chicken thighs, pineapple, white rice, **Caesar salad**, salt, cilantro, pepper, lemon, *kale* juice, garlic, olive oil, *Brussels sprouts*	361.03 (ITA)	20.01	30.60	4.63
dish_1561492204	Olive oil, blueberries, onions, tomatoes, salt, **fried rice**, chicken, pepper, mustard, garlic, bell peppers, *millet*, *jalapenos*, spinach (raw), vinegar, radishes	358.14 (ITA)	0.45	15.45	60.10
dish_1566502315	White rice, *pork*, soy sauce, *chard*, brown rice, lemon juice, garlic, parsley, olive oil, **Caesar salad**, carrot, *chive*, salt, pepper, *bok choy*, sugar, chicken, rosemary, basil	356.32 (ITA)	11.87	31.22	7.86
dish_1563984729	**Scrambled eggs**, chicken apple sausage	355.67 (ITA)	16.02	36.49	7.48
dish_1563984835	**Vinaigrette**, raspberries, blackberries, mixed greens, granola, **scrambled eggs**, *salsa*, chicken apple sausage	343.82 (ITA)	14.85	35.30	9.84
dish_1566849387	Parmesan cheese, **Caesar salad**, olives, chicken, mixed greens, avocado	336.26 (ITA)	8.51	33.04	6.44
dish_1573232499	*Bacon*, **scrambled eggs**, *broccoli*, olive oil, **fried rice**	334.52 (ITA)	2.44	21.64	43.37
dish_1568056258	Olives, carrot, chicken, **Caesar dressing**, cherry tomatoes, onions	331.70 (ITA)	7.92	43.78	6.67

Table S6 (Cont'd)

dish_1563984771	Mixed greens, raspberries, chicken apple sausage, granola blackberries, **scrambled eggs**, **vinaigrette**	324.84 (ITA)	15.29	35.16	13.34
dish_1565972674	Salt, **scrambled eggs**, chicken apple sausage, cherry tomatoes, onions, pepper, lettuce, mixed greens, lime, olive oil, vinegar, cucumbers	317.83 (ITA)	12.97	33.69	8.47
dish_1565972628	Chicken apple sausage**, scrambled eggs**	317.13 (ITA)	13.05	33.64	7.34
dish_1561492182	Spinach (raw), vinegar, radishes, **fried rice**, garlic, salt, mustard, olive oil, blueberries	315.04 (ITA)	1.68	13.05	51.63
dish_1566844766	**Caesar salad**	314.06 (ITA)	14.23	24.99	5.98
dish_1563913709	Olive oil, cucumbers, **fried rice**, **Caesar salad**, salt, **cheese pizza**, figs, cherry tomatoes, *chard*, mustard, vinegar, spinach (raw)	312.83 (ITA)	12.24	22.25	15.95

[1]Single ingredients in Nutrition5k that are actually composite recipes are indicated in bold. Ingredients in Nutrition5k without a corresponding cooked food item in the Italian FCDB are indicated in italics. [2]The tag "ITA" and "USA" at the bottom identified which nutrient is the highest one. For example, 754.00 (ITA) indicates that Italian nutritional values were higher than the corresponding US ones, and vice versa. Abbreviations: FCDB, Food Composition Database; SD, standard deviation; USDA, United States Department of Agriculture.

Table S7: Top 25 dishes showing the most extreme (i.e., >99.5%) differences in absolute value across FCDBs for protein content.

**DISH_ID**	**INGREDIENTS**	**ǀΔ Energyǀ (kcal)**	**ǀΔ Proteinsǀ (g)**	**ǀΔ Fatsǀ (g)**	**ǀΔ Carbohydratesǀ (g)**
dish_1558115364	*Potatoes*, Apples with peel, almonds, *bacon*[1]	67.42	25.82 (USA)[2]	3.63	9.26
dish_1565123881	**Caesar salad**[1], olive oil, onions, **cheese pizza**, *zucchini*, spinach (cooked), *millet*, salt, *shallots*, *bok choy*, *beef*, *chayote squash*, vinegar	562.49	24.91 (ITA)	44.72	12.27
dish_1565898659	Cherry tomatoes, *tuna*, olives, *salmon*, spinach (raw), onions, **chili**, salt, cilantro, lime, ginger, brown rice, *jalapenos*, lemon juice, olive oil, carrot, *shallots*, garlic	97.62	24.00 (USA)	24.88	4.02
dish_1565898758	*Tuna*, olives, *green beans*, spinach (raw), ginger, brown rice, *shallots*, garlic, cilantro, carrot, cherry tomatoes, *salmon*, *jalapenos*, lemon juice, olive oil, onions, salt, lime, **chili**	91.45	23.87 (USA)	24.88	7.10
dish_1565898723	*Shallots*, *green beans*, cherry tomatoes, ginger, *jalapenos*, onions, olive oil, *tuna*, garlic, lime, cilantro, *salmon*, olives, salt, brown rice, spinach (raw), lemon juice, carrot, **chili**	94.33	23.85 (USA)	24.88	6.31
dish_1558122740	Grapes, mixed greens, Apples with peel, *potatoes*, *bacon*	59.84	23.70 (USA)	3.43	12.02
dish_1566502211	**Caesar salad**	453.86	20.56 (ITA)	36.11	8.65

Table S7 (Cont'd)

dish_1558115047	Mixed greens, almonds, Apples with peel	5.87	20.16 (USA)	0.90	8.39
dish_1558380181	Almonds, apples with peel, grapes	0.18	20.15 (USA)	1.74	4.14
dish_1565204931	Onions, vinegar, chicken thighs, pineapple, white rice, **Caesar salad**, pepper, salt, lemon juice, garlic, olive oil, *Brussels sprouts*, cilantro, *kale*	361.03	20.01 (ITA)	30.60	4.63
dish_1558114284	Apples with peel, grapes, cantaloupe	15.59	19.90 (USA)	0.30	11.34
dish_1558115282	Apples with peel	11.52	19.87 USA)	0.22	13.97
dish_1558116547	Apples with peel	11.52	19.87 (USA)	0.22	13.97
dish_1558109945	Apples with peel	11.44	19.73 (USA)	0.21	13.87
dish_1558114086	Apples with peel	11.44	19.73 (USA)	0.21	13.87
dish_1558114875	Apples with peel	11.44	19.73 (USA)	0.21	13.87
dish_1558372433	Apples with peel	11.44	19.73 (USA)	0.21	13.87
dish_1558376768	Apples with peel	11.44	19.73 (USA)	0.21	13.87
dish_1558379588	Apples with peel	11.44	19.73 (USA)	0.21	13.87
dish_1558380152	Apples with peel	11.44	19.73 (USA)	0.21	13.87
dish_1558373074	Apples with peel	11.36	19.60 (USA)	0.21	13.77
dish_1558375506	Apples with peel	11.36	19.60 (USA)	0.21	13.77
dish_1560356523	*Bacon*	243.80	19.50 (USA)	16.93	1.29
dish_1564502063	*Bacon*	243.80	19.50 (USA)	16.93	1.29
dish_1564073860	*Bacon*	241.15	19.29 (USA)	16.74	1.27

[1]Single ingredients in Nutrition5k that are actually composite recipes are indicated in bold. Ingredients in Nutrition5k without a corresponding cooked food item in the Italian FCDB are indicated in italics. [2]The tag "ITA" and "USA" at the bottom identified which nutrient is the highest one. For example, 25.82 (USA) indicates that US nutritional values were higher than the corresponding Italian ones, and vice versa. Abbreviations: FCDB, Food Composition Database; SD, standard deviation; USDA, United States Department of Agriculture.

Table S8: Top 25 dishes showing the most extreme (i.e., >99.5%) differences in absolute value across FCDBs for fats content.

DISH_ID	INGREDIENTS	ǀΔ Energyǀ (kcal)	ǀΔ Proteinsǀ (g)	ǀΔ Fatsǀ (g)	ǀΔ Carbohydratesǀ (g)
dish_1567714934	Parmesan cheese, **Caesar dressing**[1], avocado, mixed greens, chicken, olives	754.00	8.32	91.53<br>(ITA)[2]	15.35
dish_1566850031	Olives, carrot, chicken, **Caesar dressing**, cherry tomatoes, onions	715.80	9.06	87.68<br>(ITA)	11.37
dish_1566246626	Feta cheese, *broccoli*[1], mixed greens, chicken, olives, **Caesar dressing**	710.47	5.77	85.51<br>(ITA)	13.51
dish_1566332268	Olives, chicken, spinach (raw), avocado, **Caesar dressing**, cucumbers	637.40	3.94	74.51<br>(ITA)	14.61
dish_1565987201	Cucumbers, spinach (raw), parmesan cheese, **Caesar dressing**, chicken, olives	589.94	7.23	71.08<br>(ITA)	10.04
dish_1565725244	Parmesan cheese, cherry tomatoes, chicken, bell peppers, carrot, olives, onions, **Caesar dressing**	380.81	8.35	50.59<br>(ITA)	13.03
dish_1565123881	**Caesar salad**, olive oil, onions, **cheese pizza**, *zucchini*, spinach (cooked), *millet*, salt, *shallots*, *bok choy*, *beef*, *chayote squash*, vinegar	562.49	24.91	44.72<br>(ITA)	12.27
dish_1568056258	Olives, carrot, chicken, **Caesar dressing**, cherry tomatoes, onions	331.70	7.92	43.78<br>(ITA)	6.67
dish_1566502271	*Chard*, parsley, *chive*, olive oil, carrot, **Caesar salad**, garlic, chicken, salt, lemon juice, basil, brown rice, rosemary	449.37	17.56	37.81<br>(ITA)	8.54
dish_1563984729	**Scrambled eggs**, chicken apple sausage	355.67	16.02	36.49<br>(ITA)	7.48

Table S8 (Cont'd)

dish_1566502211	**Caesar salad**	453.86	20.56	36.11 (ITA)	8.65
dish_1563984835	**Vinaigrette**, raspberries, blackberries, mixed greens, granola, **scrambled eggs**, *salsa*, chicken apple sausage	343.82	14.85	35.30 (ITA)	9.84
dish_1563984771	Mixed greens, raspberries, chicken apple sausage, granola blackberries, **scrambled eggs**, **vinaigrette**	324.84	15.29	35.16 (ITA)	13.34
dish_1562790855	Radishes, *fish*, cherry tomatoes, *shallots*, onions, mustard, *pork*, parsley, garlic, basil, salt, pepper, lemon juice, brown rice, bell peppers, country rice, cucumbers, olive oil, vinegar, *broccoli*, *green onions*	84.77	9.87	34.81 (USA)	2.49
dish_1567022383	Onions, **Caesar dressing**, bell peppers, carrot, cherry tomatoes, olives, chicken, parmesan cheese	242.67	7.78	34.48 (ITA)	9.93
dish_1565972674	Salt, **scrambled eggs**, chicken apple sausage, cherry tomatoes, pepper, lettuce, mixed greens, lime, olive oil, vinegar, onions, cucumbers	317.83	12.97	33.69 (ITA)	8.47
dish_1565972715	Cucumbers, vinegar, *green beans*, **scrambled eggs**, pepper, lettuce, chicken apple sausage, cherry tomatoes, lime, mixed greens, salt, olive oil, onions	312.41	13.21	33.69 (ITA)	12.24

Table S8 (Cont'd)

dish_1565972628	Chicken apple sausage, **scrambled eggs**	317.13	13.05	33.64 (ITA)	7.34
dish_1568144954	Carrot, chicken, salt, onions, **Caesar dressing**, olive oil, pepper, cherry tomatoes, olives	250.08	5.48	33.15 (ITA)	8.47
dish_1566849387	Parmesan cheese, **Caesar salad**, olives, chicken, mixed greens, avocado	336.26	8.51	33.04 (ITA)	6.44
dish_1566502315	White rice, *pork*, soy sauce, *chard*, brown rice, lemon juice, garlic, parsley, olive oil, **Caesar salad**, carrot, *chive*, salt, pepper, *bok choy*, sugar, chicken, rosemary, basil	356.32	11.87	31.22 (ITA)	7.86
dish_1565204931	Onions, vinegar, chicken thighs, pineapple, white rice, **Caesar salad**, *kale*, cilantro, pepper, salt, lemon juice, garlic, olive oil, *Brussels sprouts*	361.03	20.01	30.60 (ITA)	4.63
dish_1568144925	Olive oil, onions, salt, carrot, olives, pepper, cherry tomatoes, **Caesar dressing**	258.58	0.21	30.09 (ITA)	8.47
dish_1567801468	**Caesar salad**	375.34	17.00	29.86 (ITA)	7.15
dish_1563567372	Cherry tomatoes, *fish*, salt, wheat berry, lemon juice, *chard*, *kale*, parsley, olive oil, pizza, *shallots*, garlic, *chive*	56.13	0.83	28.83 (USA)	11.43

[1]Single ingredients in Nutrition5k that are actually composite recipes are indicated in bold. Ingredients in Nutrition5k without a corresponding cooked food item in the Italian FCDB are indicated in italics. [2]The tag "ITA" and "USA" at the bottom identified which nutrient is the highest one. For example, 91.53 (ITA) indicates that Italian nutritional values were higher than the corresponding US ones, and vice versa. Abbreviations: FCDB, Food Composition Database; SD, standard deviation; USDA, United States Department of Agriculture.

Table S9: Top 25 dishes showing the most extreme (i.e., >99.5%) differences in absolute value across FCDBs for carbohydrates content.

DISH_ID	INGREDIENTS	∣Δ Energy∣ (kcal)	∣Δ Proteins∣ (g)	∣Δ Fats∣ (g)	∣Δ Carbohydrates∣ (g)
dish_1561492228	Cherry tomatoes, blueberries, *jalapenos*[1], vinegar, pepper, chicken, parsley, salt, lemon juice, tomatoes, mustard, olive oil, *chive*, *shallots*, spinach (raw), *chard*, radishes, *millet*, bell peppers, garlic, *fish*, **fried rice**[1], onions, wheat berry	390.08	0.16	5.68	62.65 (ITA)2
dish_1561492204	Olive oil, blueberries, onions, tomatoes, salt, **fried rice**, chicken, pepper, mustard, garlic, bell peppers, *millet*, *jalapenos*, spinach (raw), vinegar, radishes	358.14	0.45	15.45	60.10 (ITA)
dish_1563389199	*Broccoli*, garlic, pepper, arugula, vinegar, *chard*, blueberries, bulgur, olive oil, pecans, mustard, *tofu*, salt, carrot	198.79	6.27	0.97	51.99 (USA)
dish_1561492182	Spinach (raw), vinegar, radishes, **fried rice**, garlic, salt, mustard, olive oil, blueberries	315.04	1.68	13.05	51.63 (ITA)
dish_1563389153	Olive oil, vinegar, arugula, *chard*, pepper, carrot, bulgur, mustard, salt, *broccoli*, pecans, garlic, blueberries	203.59	6.33	0.97	51.27 (ITA)
dish_1571932448	**Oatmeal**	291.25	7.70	6.10	51.12 (ITA)

Table S9 (Cont'd)

dish_1560801041	*Pork*, *zucchini*, arugula, chicken breast, cherry tomatoes, rosemary, lemon juice, thyme, olive oil, onions, wheat berry, parmesan cheese, pepper, spinach (raw), *cauliflower*, garlic, chickpeas, white rice, salt, bulgur, parsley, *kale*	210.31	6.50	3.67	46.07 (USA)
dish_1560800988	Pepper, *cauliflower*, chickpeas, salt, bulgur, olive oil, *kale*, lemon juice, parsley	173.12	4.37	0.94	45.93 (USA)
dish_1560801020	*Cauliflower*, garlic, spinach (raw), parmesan cheese, wheat berry, chickpeas, pepper, chicken breast, cherry tomatoes, thyme, lemon juice, olive oil, parsley, *kale*, bulgur, salt	178.20	4.51	1.36	45.83 (USA)
dish_1563909550	Cucumbers, mustard, mustard greens, olive oil, garlic, *green beans*, tomatoes, salt, **fried rice**, vinegar, spinach (raw)	295.72	1.74	12.47	45.15 (ITA)
dish_1568404889	Olive oil, vinegar, radishes, soy sauce, carrot, **fried rice**, mixed greens, garlic, *green onions*, *cabbage*, rice noodles	272.17	3.99	10.18	44.84 (ITA)
dish_1566328988	*Bok choy*, *shallots*, *beef*, vinegar, *jalapenos*, bell peppers, spinach (raw), pepper, onions, olive oil, tomatoes, garlic, **fried rice**, chicken, **cheese pizza**, salt, spinach (cooked), *millet*	218.38	1.57	8.71	44.65 (ITA)

Table S9 (Cont'd)

dish_1573849915	**Dumplings**, spinach (cooked)	36.17	2.70	14.43	44.45 (USA)
dish_1573232499	*Bacon*, **scrambled eggs**, *broccoli*, olive oil, **fried rice**	334.52	2.44	21.64	43.37 (ITA)
dish_1573849852	**Dumplings**	37.31	2.81	14.14	42.62 (USA)
dish_1563909580	Mustard greens, cucumbers, tomatoes, salt, mustard, garlic, **fried rice**, **cheese pizza**, vinegar, spinach (raw), *green beans*, olive oil	291.52	3.59	12.99	42.34 (ITA)
dish_1568404850	**Fried rice**	244.10	1.20	10.14	40.85 (ITA)
dish_1573849990	*Bacon*, onions, olive oil, **dumplings**, goat cheese, grapes, spinach (cooked), *chive*, pizza dough	43.31	0.85	13.02	40.66 (USA)
dish_1566329021	Bell peppers, *shallots*, *beef*, garlic, vinegar, spinach (raw), pepper, olive oil, tomatoes, **cheese pizza**, salt, chicken, *bok choy*, *jalapenos*, *Brussels sprouts*, onions, **fried rice**, spinach (cooked), *millet*	223.09	0.87	8.88	40.61 (ITA)
dish_1568060501	Tomatoes, cherry tomatoes, garlic, lemon juice, cucumbers, mustard greens, olive oil, parsley, rosemary, lime, **fried rice**, mangos, white rice, *pork*, *zucchini*, *jalapenos*, arugula, onions, cilantro, salt	142.17	6.47	2.32	40.52 (ITA)
dish_1564761426	*Egg whites*, **fried rice**	227.33	1.90	9.68	38.94 (ITA)

Table S9 (Cont'd)

dish_1566329049	Bell peppers, *cauliflower*, *beef*, *jalapenos*, spinach (raw), *Brussels sprouts*, onions, olive oil, pepper, garlic, salt, *shallots*, spinach (cooked), chicken, *bok choy*, *millet*, vinegar, **cheese pizza**, tomatoes, **fried rice**	227.54	0.38	8.80	38.56 (ITA)
dish_1564761457	**Fried rice**, *turkey bacon*, egg whites	205.55	0.90	8.73	38.29 (ITA)
dish_1563897870	**Oatmeal**	217.13	5.74	4.54	38.11 (ITA)
dish_1562603895	Blackberries, mixed greens, **vinaigrette**, **hash browns**, **scrambled eggs**, raspberries, granola, sausage	167.30	3.25	4.76	37.53 (USA)

[1]Single ingredients in Nutrition5k that are actually composite recipes are indicated in bold. Ingredients in Nutrition5k without a corresponding cooked food item in the Italian FCDB are indicated in italics. [2]The tag "ITA" and "USA" at the bottom identified which nutrient is the highest one. For example, 62.65 (ITA) indicates that Italian nutritional values were higher than the corresponding US ones, and vice versa. Abbreviations: FCDB, Food Composition Database; SD, standard deviation; USDA, United States Department of Agriculture.

DISH_ID	INGREDIENT_ID	Ingredient name	Ingredient mass	BDA food item	Energy (kcal)	Energy (kJ)	Total protein (g)	Animal protein (g)	Vegetable protein (g)	Total fat (g)	Animal fat (g)	Vegetable fat (g)	Cholesterol (g)
dish_1574711589	ingr_0000000023	**brown rice**	5.65	RICE, BROWN, WHOLEGRAIN, cooked	6.39	27.08	0.14	0.00	0.14	0.03	0.00	0.03	0.00
	ingr_0000000027	**broccoli**	52.62	BROCCOLI, PURPLE SPROUTING	17.37	73.67	1.58	0.00	1.58	0.21	0.00	0.21	0.00
	ingr_0000000073	**radishes**	1.13	RED RADISH	0.15	0.63	0.01	0.00	0.01	0.00	0.00	0.00	0.00
	ingr_0000000122	**garlic**	0.14	GARLIC, fresh	0.06	0.27	0.00	0.00	0.00	0.00	0.00	0.00	0.00
	ingr_0000000155	**chicken thighs**	56.54	CHICKEN, LEG-LOWER PART, with skin, cooked, oven	113.65	477.76	17.64	17.64	0.00	4.81	4.81	0.00	51.45
	ingr_0000000161	**olive oil**	4.07	VEGETABLE OIL, OLIVE, EXTRAVIRGIN	36.60	150.47	0.00	0.00	0.00	4.07	0.00	4.07	0.00
	ingr_0000000174	**bread crumbs**	0.73	BREADCRUMBS	2.59	11.02	0.07	0.00	0.07	0.02	0.00	0.02	0.00
	ingr_0000000189	**vinegar**	0.57	VINEGAR	0.02	0.09	0.00	0.00	0.00	0.00	0.00	0.00	0.00
	ingr_0000000243	**mozzarella cheese**	10.08	MOZZARELLA CHEESE, COW	25.49	105.90	1.88	1.88	0.00	1.96	1.96	0.00	4.64
	ingr_0000000291	**salt**	0.08	SALT	0.00	0.00	0.00	0.00	0.00	0.00	0.00	0.00	0.00
	ingr_0000000448	**jalapenos**	0.66	PEPPERS, CHILI, RED	0.20	0.82	0.01	0.00	0.01	0.00	0.00	0.00	0.00
	ingr_0000000511	**spinach (cooked)**	32.00	SPINACH, canned	8.00	33.28	0.90	0.00	0.90	0.16	0.00	0.16	0.00
	ingr_0000000515	**arugula**	1.41	ARUGULA OR ROCKET	0.40	1.64	0.04	0.00	0.04	0.01	0.00	0.01	0.00
	ingr_0000000518	**thyme**	0.28	THYME, dried ground	0.88	3.68	0.03	0.00	0.03	0.02	0.00	0.02	0.00
	ingr_0000000520	**lemon juice**	0.07	JUICE, LEMON, fresh	0.00	0.02	0.00	0.00	0.00	0.00	0.00	0.00	0.00
	ingr_0000000521	**basil**	2.80	BASIL, fresh	1.37	5.77	0.09	0.00	0.09	0.02	0.00	0.02	0.00
	ingr_0000000524	**parsley**	0.28	PARSLEY, dried	0.76	3.16	0.04	0.00	0.04	0.02	0.00	0.02	0.00

Figure S1. Nutrition5k dataset in the long format, with each ingredient matched with the corresponding food item from the Italian FCDB and the corresponding Italian FCDB-specific nutritional composition. Abbreviations: FCDB, Food Composition Database.

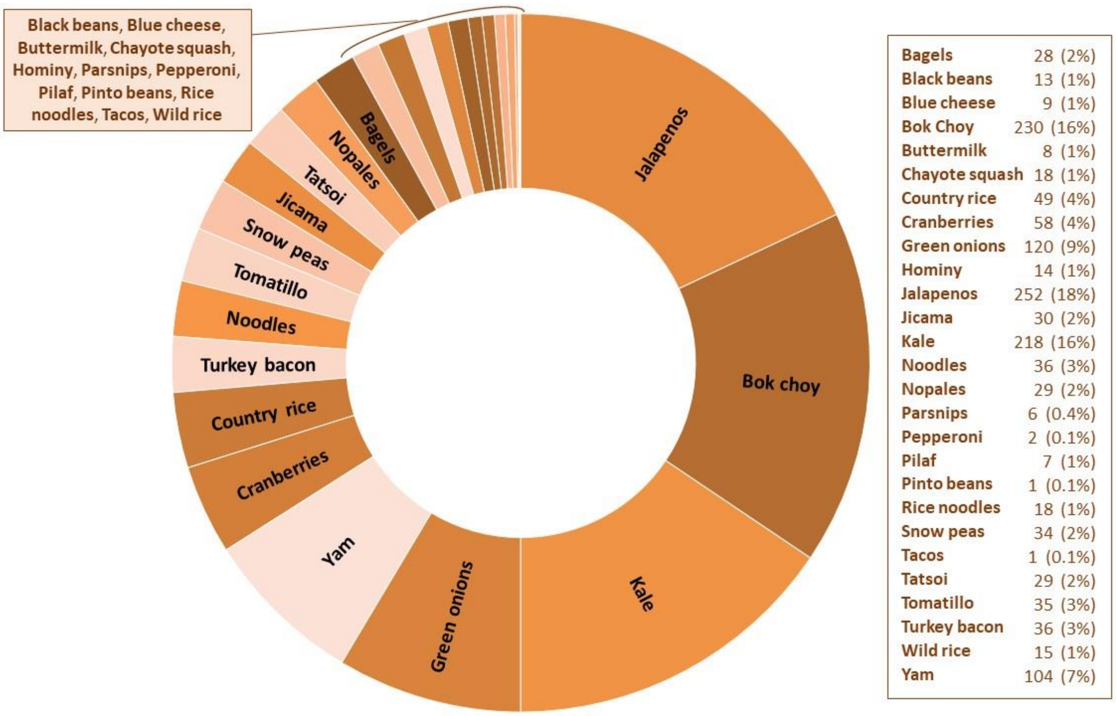

Figure S2. Imputation of ingredients from Nutrition5k by similarity: frequency of specific ingredients.

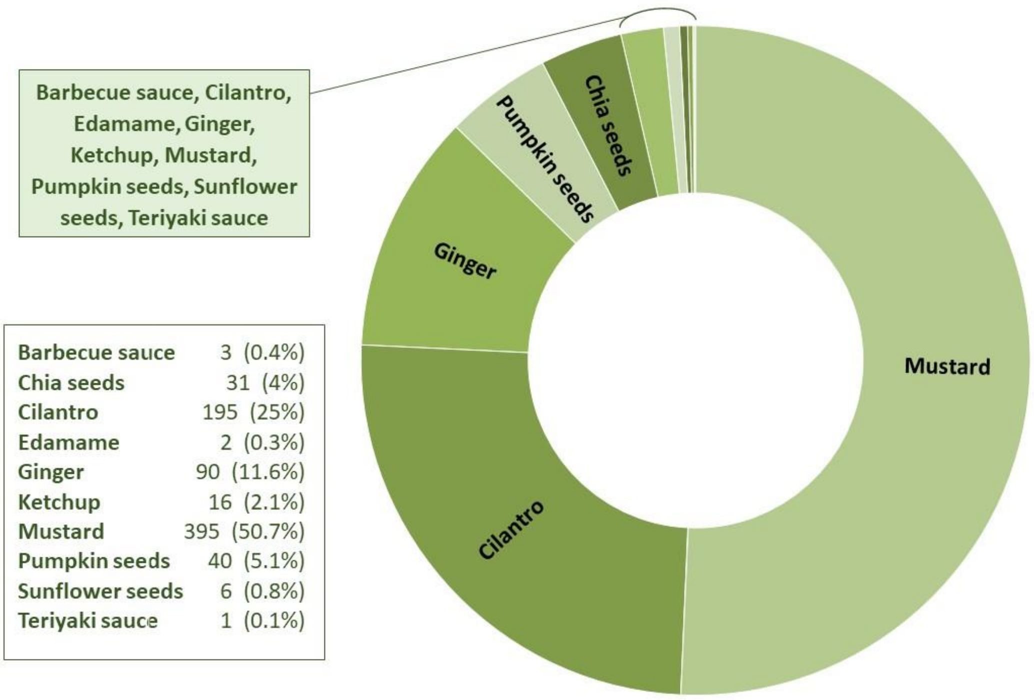

Figure S3. Imputation of ingredients from Nutrition5k by international sources: frequency of specific ingredients.

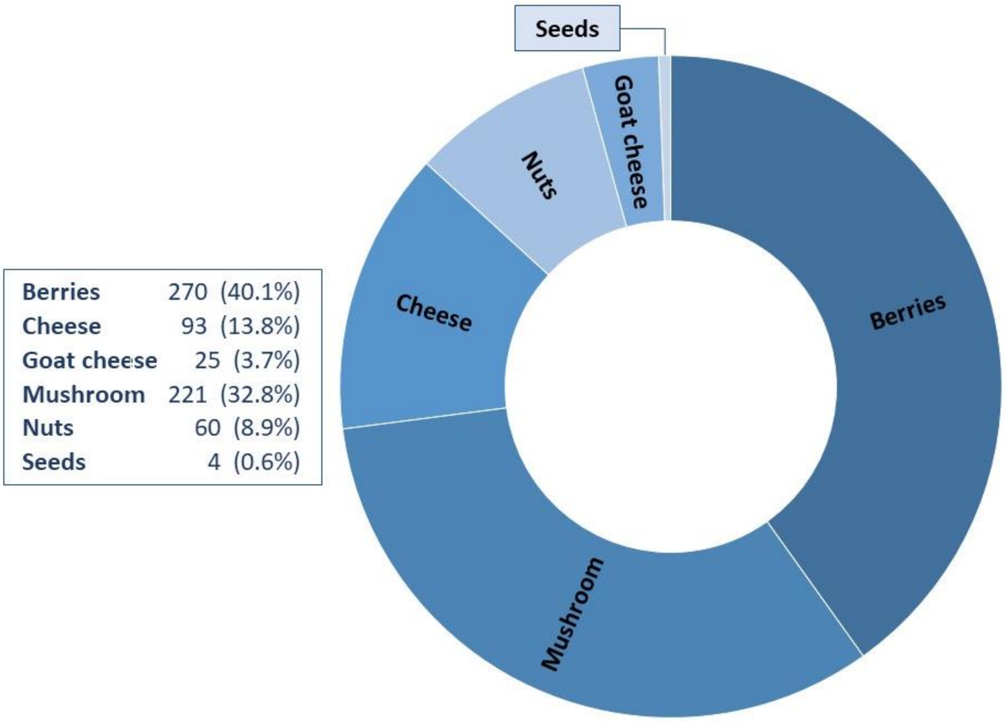

Figure S4. Imputation of ingredients from Nutrition5k by group of related food items: frequency of specific ingredients.

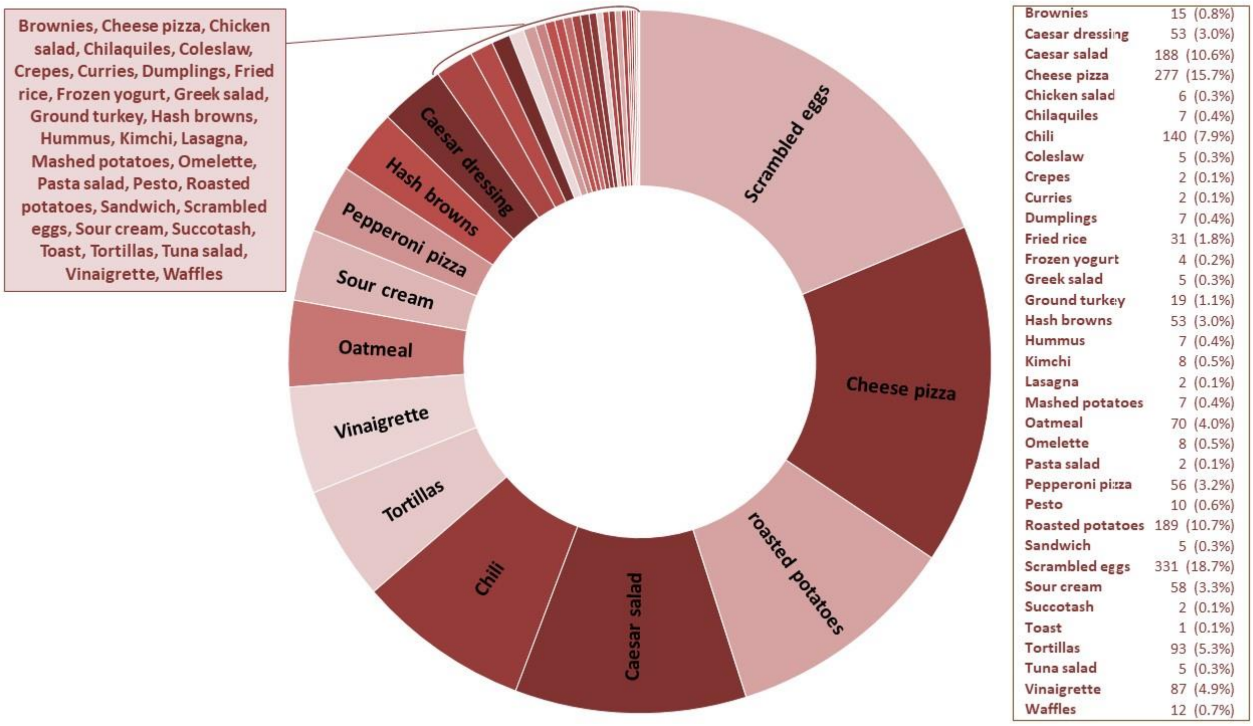

Figure S5. Imputation of ingredients from Nutrition5k by creation of composite recipes: frequency of specific ingredients.

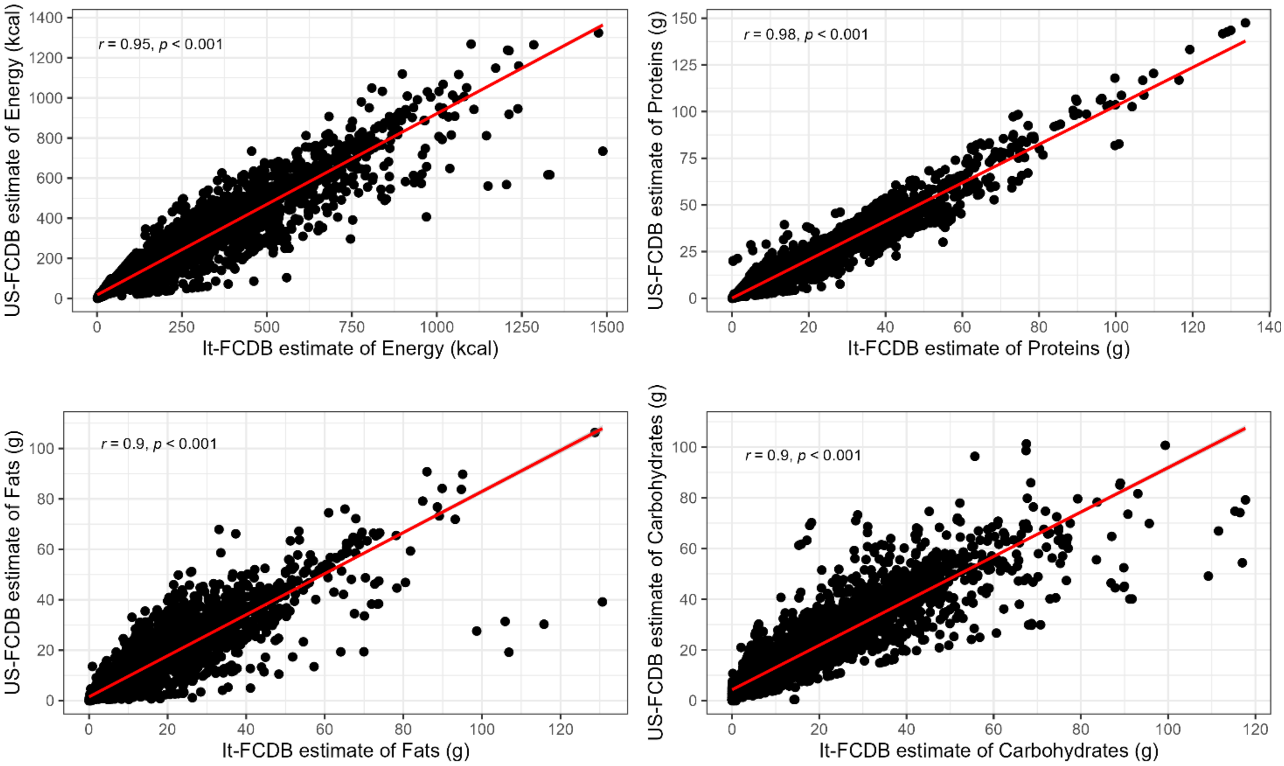

Figure S6. Scatterplots of nutrient content obtained across Italian and US FCDBs . The Pearson correlation coefficients are indicated with the "r" letter. The corresponding p-values from the statistical test on Pearson correlation coefficients are indicated aside. Abbreviations: FCDB, Food Composition Database.

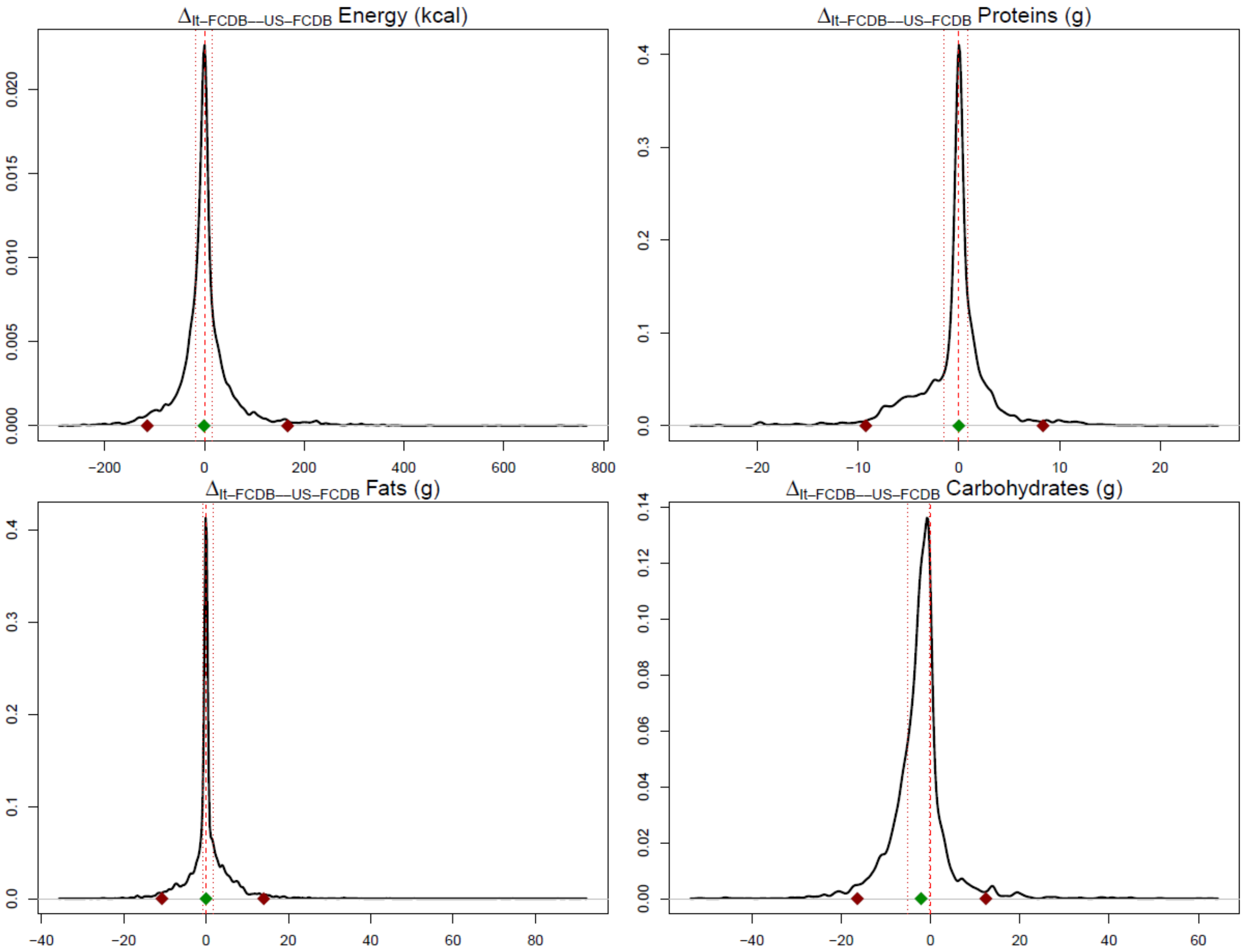

Figure S7. Kernel density estimation plots of the difference between Italian and US FCDBs for energy and macronutrients. The dashed line indicates the reference value 0, and the dotted lines indicate the 1st and 3rd quartiles. The green diamond indicates the median value, and the red diamonds indicate the 2.5th and 97.5th percentiles. Abbreviations: FCDB, Food Composition Database.
